# Dynamics of Gene Loss following Ancient Whole-Genome Duplication in the Cryptic *Paramecium* Complex

**DOI:** 10.1093/molbev/msad107

**Published:** 2023-05-08

**Authors:** Jean-Francois Gout, Yue Hao, Parul Johri, Olivier Arnaiz, Thomas G Doak, Simran Bhullar, Arnaud Couloux, Fréderic Guérin, Sophie Malinsky, Alexey Potekhin, Natalia Sawka, Linda Sperling, Karine Labadie, Eric Meyer, Sandra Duharcourt, Michael Lynch

**Affiliations:** Department of Biology, Indiana University, Bloomington, IN; Biodesign Center for Mechanisms of Evolution, Arizona State University, Tempe, AZ; Department of Biological Sciences, Mississippi State University, Starkville, MS; Biodesign Center for Mechanisms of Evolution, Arizona State University, Tempe, AZ; Cancer and Cell Biology Division, Translational Genomics Research Institute, Phoenix, AZ; Department of Biology, Indiana University, Bloomington, IN; Biodesign Center for Mechanisms of Evolution, Arizona State University, Tempe, AZ; School of Life Sciences, Arizona State University, Tempe, AZ; Institute for Integrative Biology of the Cell (I2BC), Commissariat à l'Energie Atomique (CEA), CNRS, Université Paris-Sud, Université Paris-Saclay, Gif-sur-Yvette, France; Department of Biology, Indiana University, Bloomington, IN; National Center for Genome Analysis Support, Indiana University, Bloomington, IN; Institut de biologie de l’ENS, Département de Biologie, Ecole Normale Supérieure, CNRS, Inserm, Université PSL, Paris, France; Génomique Métabolique, Genoscope, Institut François Jacob, Commissariat à l'Energie Atomique (CEA), CNRS, Univ Evry, Université Paris-Saclay, Evry, France; Université Paris Cité, CNRS, Institut Jacques Monod, Paris, France; Institut de biologie de l’ENS, Département de Biologie, Ecole Normale Supérieure, CNRS, Inserm, Université PSL, Paris, France; Department of Microbiology, Faculty of Biology, Saint Petersburg State University, Saint Petersburg, Russia; Laboratory of Cellular and Molecular Protistology, Zoological Institute RAS, Saint Petersburg, Russia; Institute of Systematics and Evolution of Animals, Polish Academy of Sciences, Krakow, Poland; Institute for Integrative Biology of the Cell (I2BC), Commissariat à l'Energie Atomique (CEA), CNRS, Université Paris-Sud, Université Paris-Saclay, Gif-sur-Yvette, France; Genoscope, Institut François Jacob, Commissariat à l'Energie Atomique (CEA), Université Paris-Saclay, Evry, France; Institut de biologie de l’ENS, Département de Biologie, Ecole Normale Supérieure, CNRS, Inserm, Université PSL, Paris, France; Université Paris Cité, CNRS, Institut Jacques Monod, Paris, France; Department of Biology, Indiana University, Bloomington, IN; Biodesign Center for Mechanisms of Evolution, Arizona State University, Tempe, AZ

**Keywords:** *Paramecium aurelia*, whole genome duplication, gene retention, gene loss, ohnologs, orthologs

## Abstract

Whole-genome duplications (WGDs) have shaped the gene repertoire of many eukaryotic lineages. The redundancy created by WGDs typically results in a phase of massive gene loss. However, some WGD–derived paralogs are maintained over long evolutionary periods, and the relative contributions of different selective pressures to their maintenance are still debated. Previous studies have revealed a history of three successive WGDs in the lineage of the ciliate *Paramecium tetraurelia* and two of its sister species from the *Paramecium aurelia* complex. Here, we report the genome sequence and analysis of 10 additional *P. aurelia* species and 1 additional out group, revealing aspects of post-WGD evolution in 13 species sharing a common ancestral WGD. Contrary to the morphological radiation of vertebrates that putatively followed two WGD events, members of the cryptic *P. aurelia* complex have remained morphologically indistinguishable after hundreds of millions of years. Biases in gene retention compatible with dosage constraints appear to play a major role opposing post-WGD gene loss across all 13 species. In addition, post-WGD gene loss has been slower in *Paramecium* than in other species having experienced genome duplication, suggesting that the selective pressures against post-WGD gene loss are especially strong in *Paramecium*. A near complete lack of recent single-gene duplications in *Paramecium* provides additional evidence for strong selective pressures against gene dosage changes. This exceptional data set of 13 species sharing an ancestral WGD and 2 closely related out group species will be a useful resource for future studies on *Paramecium* as a major model organism in the evolutionary cell biology.

## Introduction

Gene duplication is a common type of genomic alteration that can occur at frequencies rivaling that of point mutations (Lynch 2007; Lipinski et al. 2011; Schrider et al. 2013; Reams and Roth 2015). Because duplicated genes are often redundant, mutations crippling one copy are expected to frequently drift to fixation, unaffected by selection. As a consequence, the fate of most duplicated genes is rapid pseudogenization and eventual evolution beyond recognition. However, some ancient duplicated genes are ubiquitously retained in the genomes of all free-living organisms sequenced to date (Zhang 2003). Therefore, selective pressures opposing the loss of genes generated by WGD must be commonly operating despite the initial redundancy between the two copies.

Several models have been proposed to explain the long-term retention of duplicated genes. Retention can happen through change in function when one copy acquires mutations conferring a new beneficial function (neofunctionalization; Ohno 1970) or when each copy independently loses a subset of the functions performed by the ancestral (preduplication) gene (subfunctionalization; Force et al. 1999; Lynch and Force 2000). Additionally, duplicated genes can also be retained without a change in their function, as when dosage constraints drive selection to maintain the total required amount of transcripts summed over both copies (Edger and Pires 2009; Birchler and Veitia 2012).

In its most extreme form, duplication can encompass the entire genome, creating a new copy of each gene. Such whole-genome duplication (WGD) events are common, with evidence of ancient WGDs in the lineages of many eukaryotes, including the budding yeast (Kellis et al. 2004), insects (Li et al. 2018), the African clawed frog (Session et al. 2016), salmonids (Berthelot et al. 2014), and *Paramecium* (Aury et al. 2006). It is also now widely accepted that two successive rounds of WGDs occurred in the ancestor of vertebrates (Hokamp et al. 2003; Dehal and Boore 2005; Holland and Ocampo Daza 2018) and that an additional round of genome duplication arose in the lineage leading to all teleost fish (Meyer and Schartl 1999; Jaillon et al. 2004; Howe et al. 2013; Glasauer and Neuhauss 2014; Conant 2020). Additionally, WGDs are extremely common in land plants, to the point that all angiosperms are believed to have experienced at least one round of genome duplication in their history (De Bodt et al. 2005; Ren et al. 2018). Because they create the opportunity for thousands of genes to evolve new functions, WGDs have been suggested to be responsible for the evolutionary success of several lineages (De Bodt et al. 2005; Glasauer and Neuhauss 2014). However, the precise link between WGDs and evolutionary diversification remains unclear (Clarke et al. 2016; Laurent et al. 2017).

Here, we investigate the evolutionary trajectories of duplicated genes across multiple *Paramecium* species with a common ancestral WGD. The initial sequencing of the *Paramecium tetraurelia* genome revealed a history of three successive WGDs (Aury et al. 2006). Similar to what was observed in other lineages having experienced WGDs, the *Paramecium* WGDs were followed by phases of gene loss and only a fraction of WGD–derived paralogs (ohnologs) have been retained in two copies. Still, about 50% of ohnologs from the most recent WGD are retained in two copies in *P. tetraurelia* (Aury et al. 2006), a situation very different from that in the budding yeast (about 10% retention rate; Scannell et al. 2007), the other widely studied unicellular eukaryote with an ancestral WGD. Taking into consideration that the *Paramecium* WGD is estimated to be older than that for the yeast WGD (320 My vs. 100–200 My; McGrath, Gout, Johri, et al. 2014; Gordon et al. 2009), this situation makes *Paramecium* an ideal model organism for studying the earlier stages of post-WGD genome evolution.

Interestingly, the most recent *Paramecium* WGD shortly predates the first speciation events in the formation of the *Paramecium aurelia* group of 15 cryptic *Paramecium* species (Aury et al. 2006; McGrath, Gout, Doak, et al. 2014; McGrath, Gout, Johri, et al. 2014). It has been suggested that reciprocal gene losses following genome duplication have fueled, or at least enforced, the speciation of *P. aurelia* (Aury et al. 2006; McGrath, Gout, Johri, et al. 2014). However, unlike the kinds of major phenotypic innovations suggested for post-WGD plants (van de Peer et al. 2009; Edger et al. 2015) and vertebrates (Voldoire et al. 2017; Clark and Donoghue 2018), the WGD events in *Paramecium* were followed by morphological stasis. In this regard, the *Paramecium* WGD is more similar to what is observed in yeast, where species that share the WGD with *Saccharomyces cerevisiae* are morphologically very similar, although some differences exist such as cell size (Andersson and Cohn 2017), and the WGD has been linked to several important phenotypic innovations (Huminiecki and Conant 2012). The 15 species in the *P. aurelia* complex are morphologically so similar to each other that they were once thought to be members of a single species (*P. aurelia*) until Sonneborn (1937) discovered the existence of mating types and realized that the species he was studying was in fact a complex of many genetically isolated species. These observations suggest that neofunctionalization probably did not play a major role in the retention of ohnologs following the most recent genome duplication in *Paramecium*. Our previous studies based on three *P. aurelia* genomes and one pre-WGD out group pointed to an important role of dosage constraint in the retention pattern of ohnologs in *Paramecium* (McGrath, Gout, Doak, et al. 2014; McGrath, Gout, Johri, et al. 2014; Gout and Lynch 2015).

We sought to increase our understanding of post-WGD genome evolution by sequencing the somatic (macronucleus) genomes of the remaining *P. aurelia* species and mapping the trajectories of all genes created by the recent WGD and the subsequent speciation events in *P. aurelia*. We investigated the different gene conversion patterns within the two major *P. aurelia* subclades. We also generated transcriptomic data for each species in order to characterize gene expression levels and better understand the role of expression level and dosage constraints in ohnolog retention. The annotated genomes have been uploaded to the *Paramecium*DB (https://Paramecium.i2bc.paris-saclay.fr). With this data set, we provide the scientific community with resources comparable with those available in the budding yeast (Byrne and Wolfe 2005), thereby establishing *Paramecium* as another model organism for the study of post-WGD genome evolution.

## Results

### Genome and Transcriptome Sequence of 13 *P. aurelia* Species Sharing a Common Ancestral WGD

Previous studies have revealed a history of WGDs in the lineage of *Paramecium* species belonging to the *P. aurelia* complex (Aury et al. 2006; McGrath, Gout, Doak, et al. 2014; McGrath, Gout, Johri, et al. 2014), a group of species thought to have speciated shortly following the most recent *Paramecium* WGD. To further understand the evolutionary trajectories of WGD–derived paralogs (ohnologs), we sought to complete our previous efforts (McGrath, Gout, Doak, et al. 2014; McGrath, Gout, Johri, et al. 2014) and sequenced the remaining species from the *P. aurelia* group as well as an additional closely related out group. The complete data set contains 13 species from the *P. aurelia* group and 2 out groups that diverged before the most recent WGD: *Paramecium caudatum* and *Paramecium multimicronucleatum*. All genomes (including previously published ones) were annotated using the EuGene pipeline (Foissac et al. 2008; Arnaiz et al. 2017), and evidence for a recent WGD was observed in all species of the *P. aurelia* group but absent from both out group species (Materials and Methods). The fraction of ohnolog pairs that maintained both genes intact varied from 0.39 (*Paramecium tredecaurelia*) to 0.58 (*Paramecium jenningsi*) with a median retention level across species of 0.52 (supplementary table S1, Supplementary Material online).

### Phylogeny of the *P. aurelia* Complex

**Fig. 1.**
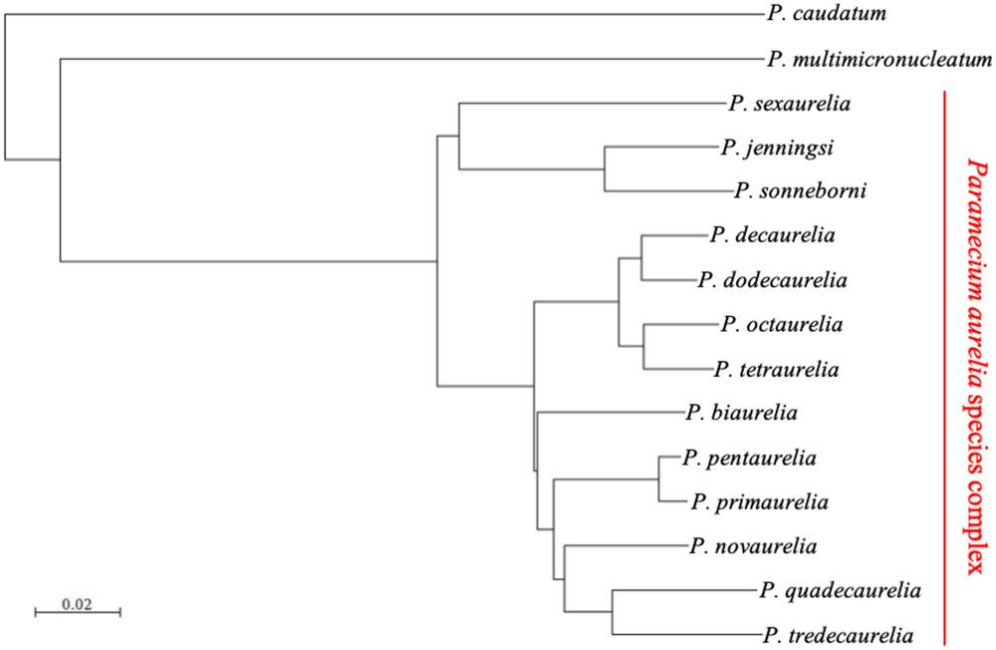
Phylogenetic tree of 13 species from the *P. aurelia* complex and 2 out group species. The tree is based on alignment of protein-coding sequences for orthologous nuclear genes present in at least half of the *P. aurelia* species sampled (21,720 sites). The tree was built using the distance method implemented in SeaView (Gouy et al. 2010). Distance is in mean number of amino acid substitutions per site.

To investigate the fates of duplicated genes, we mapped all orthologous and paralogous relationships in the *P. aurelia* complex. Because the first speciation events occurred very shortly after the most recent genome duplication, discriminating orthologs from paralogs in the most divergent *P. aurelia* species is challenging. We used PoFF (Lechner et al. 2014) to infer orthology relationships and took advantage of the low rate of large-scale genomic rearrangements in *Paramecium* to assign orthology by blocks of conserved synteny (Materials and Methods). Estimating orthology relationships in blocks of genes yields more phylogenetic signal for each orthology assignment and increases our capacity to accurately discriminate orthologs from paralogs in deep species comparisons. The final orthology assignments were used to build a reliable phylogeny of the *P. aurelia* group (fig. 1). The tree topology is similar to what was reported before (Sellis et al. 2021). All positions are strongly supported (100%) by bootstrapping, with the exception of *Paramecium biaurelia* (60%).

### Slow Post-WGD Gene Losses in Paramecium in Comparison with Other Species

**Fig. 2.**
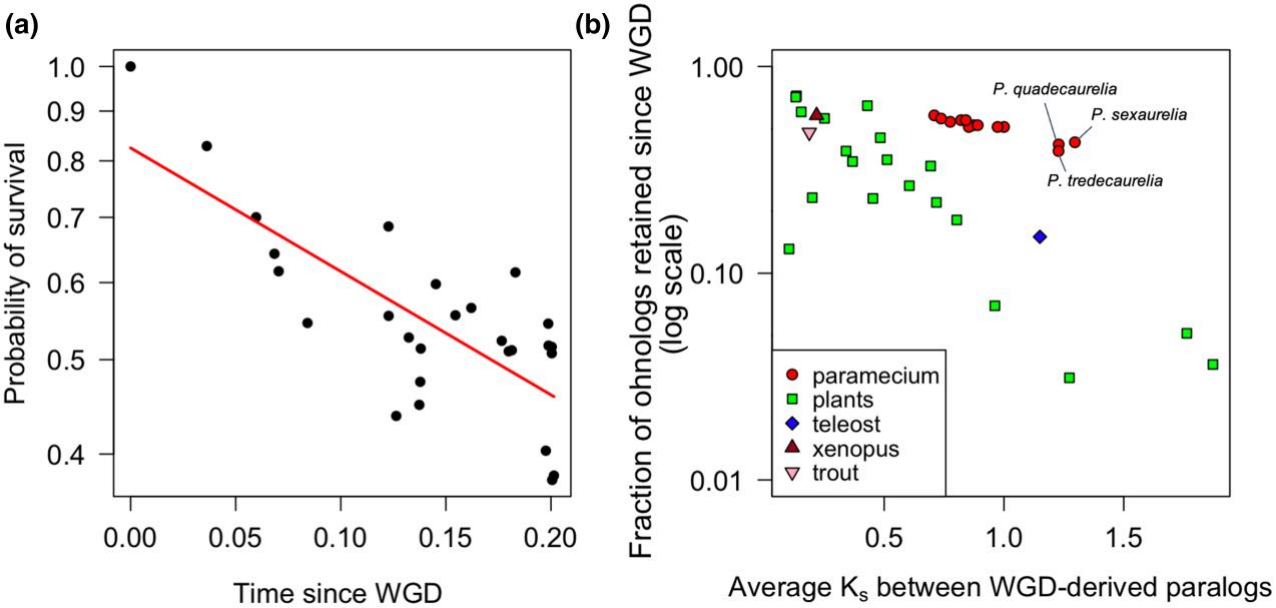
(*a*) Loss of WGD–derived paralogs in *P. aurelia* over time follows an exponential decay. We used the average amount of synonymous substitutions between the retained ohnologs as a proxy for time since the most recent genome duplication. Ancestral retention/loss rates were estimated at each node in the tree using a parsimony-based algorithm and plotted as a function of the distance between the corresponding node and the most common ancestor of all *P. aurelia* species (which coincides with the most recent WGD; McGrath, Gout, Johri, et al. 2014). (*b*) Post-WGD gene retention as a function of sequence divergence (the number of nonsynonymous substitutions per nonsynonymous site, *K*_s_) between the remaining pairs of WGD–derived paralogs in *P. aureli* and for other eukaryotes having experienced ancestral WGDs, including plants (Ren et al. 2018), teleost (Jaillon et al. 2004), salmonid (Berthelot et al. 2014), and *Xenopus* (Session et al. 2016).

We used the patterns of gene presence/absence in the established phylogeny for the 13 *P. aurelia* species to infer the timing of gene loss. Using a parsimony-based method to map the location of gene losses onto the *P. aurelia* phylogeny (Materials and Methods), we found that the proportion of surviving genes since the most recent WGD follows an exponential decay over time (fig. 2*a*). This pattern is similar to what has been observed in other eukaryotes such as yeast, teleost fish, and plants (Maere et al. 2005; Scannell et al. 2006; Inoue et al. 2015; Ren et al. 2018), although the exact shape of the survival curve is disputed (Inoue et al. 2015). We found no statistical support for a two-phase model for *Paramecium* gene loss, though such a model has been reported in teleost fish (Inoue et al. 2015). The fact that the exponential decay model fits the data suggested that the speeds at which ohnologs are lost in different eukaryotic lineages follow approximately constant rates per unit time (Nei and Roychoudhury 1973; Lynch and Conery 2000). We found 146 genes that have been lost in *Paramecium primaurelia* while still being retained in 2 copies in the closely related sister species *Paramecium pentaurelia*, highlighting the fact that gene loss is still an active ongoing process in *Paramecium*.

Having determined the general pattern of post-WGD gene loss with time, we compared the strength of selective pressures responsible for ohnolog retention in different lineages. If the *P. aurelia* speciation explosion occurred after the most recent *Paramecium* WGD (Aury et al. 2006), then the amount of time elapsed since the genome duplication will be the same in all extant species. The mutation rate in *P. aurelia* species is extremely low (Long et al. 2018), but the effective population size, generation time, and strength of selection might vary between *P. aurelia* species, resulting in different evolutionary dynamics of post-WGD ohnologs across these lineages. For each *P. aurelia* species, the fraction of ohnologs retaining both copies after the WGD was calculated. We then used the average amount of synonymous substitutions between the retained ohnologs as a proxy for time since the genome duplication. Within extant *P. aurelia* species, there is a strong negative correlation between the probability of ohnolog retention and the level of synonymous sequence divergence between the remaining pairs of ohnologs (*r* = −0.96, *P* < 0.01; fig. 2*b*, red circles). This correlation remained significant when accounting for the phylogenetic nonindependence of the data (*r* = −0.75, *P* = 0.003). Application of the same analysis to non-*Paramecium* lineages having experienced a genome duplication (Jaillon et al. 2004; Scannell et al. 2007; Berthelot et al. 2014; Session et al. 2016; Ren et al. 2018) reveals that the rate of gene loss per synonymous substitution is lower in *Paramecium* than in other phylogenetic groups (fig. 2*b*). The yeast WGD was not included in figure 2*b* because the *K*_s_ between WGD–derived ohnologs in *S. cerevisiae* (with a retention rate of ∼12%) is highly saturated (Casola et al. 2012). Supplementary Figure S1, Supplementary Material online, shows the same plot with yeast data included. It is worth pointing out that the plant WGDs were younger events comparing with the *Paramecium* WGD, ranging from 70 Ma to a few million years old (Ren et al. 2018). Thus, we interpret this observation as evidence that the strength of selection opposing gene loss is stronger in *Paramecium* than in plants and vertebrates.

### Selective Pressures Opposing Gene Loss

**Fig. 3.**
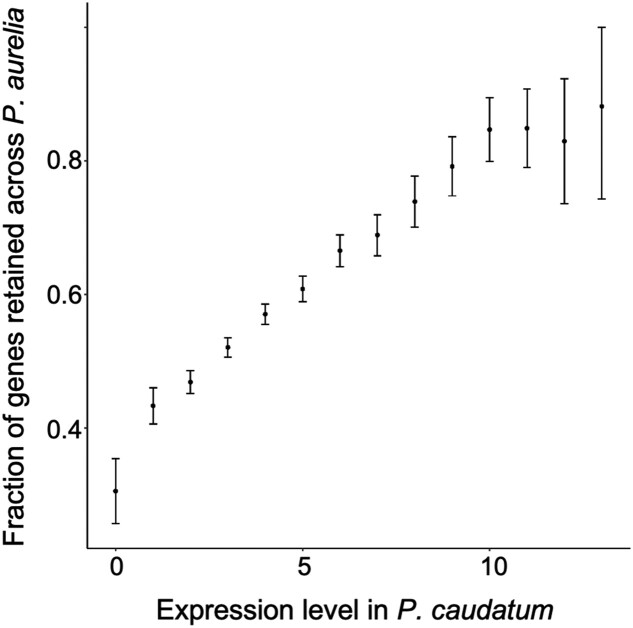
Fraction of genes retained across *P. aurelia* species as a function of ortholog average expression level log(FPKM + 0.1) in *P. caudatum*. *P. caudatum* genes were classified into 14 bins according to their expression level. For each *P. caudatum* gene with an ortholog in at least one *P. aurelia* species, a retention rate was computed as the number of *P. aurelia* species where both copies have been retained divided by the number of *P. aurelia* species with at least one ortholog for this gene. Average retention rates were computed for each bin alongside the 95% confidence interval.

To understand why selection against gene loss is stronger in *Paramecium* than in other species, we must first clarify the nature of the selective pressures promoting ohnolog retention. Although it is difficult to pinpoint which scenario (neo/subfunctionalization, or dosage constraint) is responsible for the retention of each ohnolog pair, some general trends can be derived from genome-wide analyses. We previously reported that the probability of retention is positively correlated with the expression level of ohnologs in *Paramecium* and have interpreted this observation as evidence for stronger dosage constraints in highly expressed genes (Gout et al. 2010; Gout and Lynch 2015). A similar trend had been reported for *S. cerevisiae* (Seoighe and Wolfe 1999), suggesting a universal role for expression level in post-WGD gene retention. We also confirmed that the increased retention rate for highly expressed genes was a universal pattern, present in all 13 *P. aurelia* species (supplementary fig. S2, Supplementary Material online). With 13 *P. aurelia* species available, we were able to compute a cross-species ohnolog retention rate, using the expression level of the orthologous gene in *P. caudatum* as a proxy for the preduplication expression level. As expected, we found a positive trend of the cross-species retention rate along with the expression level (fig. 3). The most highly expressed genes are much more likely to be retained in a *P. aurelia* species than the genes with low expression.

Previous studies reported a bias in the probability of post-WGD retention for different functional categories (Seoighe and Wolfe 1999; Maere et al. 2005; McGrath, Gout, Doak, et al. 2014; McGrath, Gout, Johri, et al. 2014; Rody et al. 2017). After assigning Gene Ontology (GO) terms to genes in the *P. aurelia* complex and the two out group species using the Panther pipeline (Mi et al. 2013), we found that the retention biases per GO category were highly conserved among these species. When comparing the average retention rate for each GO category between the two groups of species that diverged the earliest (*Paramecium sexaurelia*, *Paramecium jenningsi*, and *Paramecium sonneborni* vs. every other species), we found a striking positive correlation (*r* = 0.85, *P* < 0.01) between the two groups, suggesting that the different selective pressures associated with each functional category have been preserved throughout the evolution of the *P. aurelia* complex. Although the different average expression levels for each functional category explain part of this pattern (e.g. genes annotated as “structural constituent of the ribosome—GO:0003735” tend to be highly expressed and therefore are preferentially retained in two copies), we still find a number of functional categories with either significant excess or scarcity of post-WGD retention when expression level is taken into account (supplementary table S2, Supplementary Material online). One possible explanation for this pattern is that functional categories that are enriched for protein-coding genes encoding subunits of multimeric protein complexes (such as the ribosome) are preferentially retained due to increased dosage balance constraints on these genes.

### Increased Predetermination of Paralog Fate over Evolutionary Time

The previous observations suggest that the fate of ohnologs is at least partially predetermined at the time of duplication by their expression level and functional category (Gout et al. 2010; Johri et al. 2022). Although this allows us to predict which pairs of ohnologs are most likely to rapidly lose a copy, it does not inform us as to which copy, if any, is more likely to be lost. To investigate the extent of asymmetrical gene loss and its evolution with time, we estimated the fraction of parallel and reciprocal gene loss at different points on the *P. aurelia* phylogeny. Parallel gene losses are cases where two species independently lose the same copy in a pair of ohnologs. Reciprocal losses arise when two species lose a different copy in a pair of ohnologs. Gene losses that happened shortly after the genome duplication are equally distributed between reciprocal and parallel losses, as expected if both copies in a pair of ohnologs are equally likely to be eventually lost. However, the fraction of gene losses experiencing parallel losses increases with the distance between the genome duplication and the time of speciation between the two species considered (*r* = 0.40, *P* < 0.001). In other words, one of the two genes in a pair of ohnologs becomes gradually more likely to be the one that will eventually be lost (Johri et al. 2022). This observation suggests that ohnologs gradually accumulate mutations that set the two copies on different trajectories, one with increased vulnerability to eventual loss.

We previously reported that drift in expression level between ohnologs can result in a pattern such that the copy with the lowest expression is more likely to be rapidly lost (Gout and Lynch 2015). With 13 species available, we confirm that this pattern is universal across the *P. aurelia* lineage. Indeed, we found that genes in one species that are orthologous to low-expression genes in another species have higher probability of post-WGD loss (supplementary table S3, Supplementary Material online). Additionally, gene loss is biased toward the ortholog of the copy with the lowest expression level in the sister species, a bias that becomes stronger when looking at closely related species (supplementary table S3, Supplementary Material online). For example, when looking specifically at ohnologs that have been retained in *Paramecium decaurelia*, we find that only 3% of the orthologous pairs in *Paramecium dodecaurelia* (the most closely related species in our data set) have lost a copy. However, among the *P. decaurelia* ohnologs that have divergent expression level (top 5% most divergent pairs), in 22% of cases, one of the orthologs has been lost in *P. dodecaurelia*. This significant increase in probability of gene loss (*P* < 0.001, χ^2^ test) is driven by the tendency of the lost copy to be orthologous to the lowly expressed copy in the species harboring both ohnologs (82% of the cases vs. 50% expected by chance, *P* < 0.001; one-sample proportions test with continuity correction). Therefore, it appears that divergence in gene expression between ohnologs sets the two copies on opposite trajectories for their long-term survival. However, contrary to our previous prediction (Gout and Lynch 2015), we did not find any evidence for compensatory mutations increasing the expression level of the remaining copy. Therefore, it is possible that decreased expression level in one copy is a simple consequence of reduced dosage requirements, rather than being a reflection of compensatory increased expression level in the other copy.

### Subsampling Gene Trees to Infer the Placement of WGD(s)

**Fig. 4.**
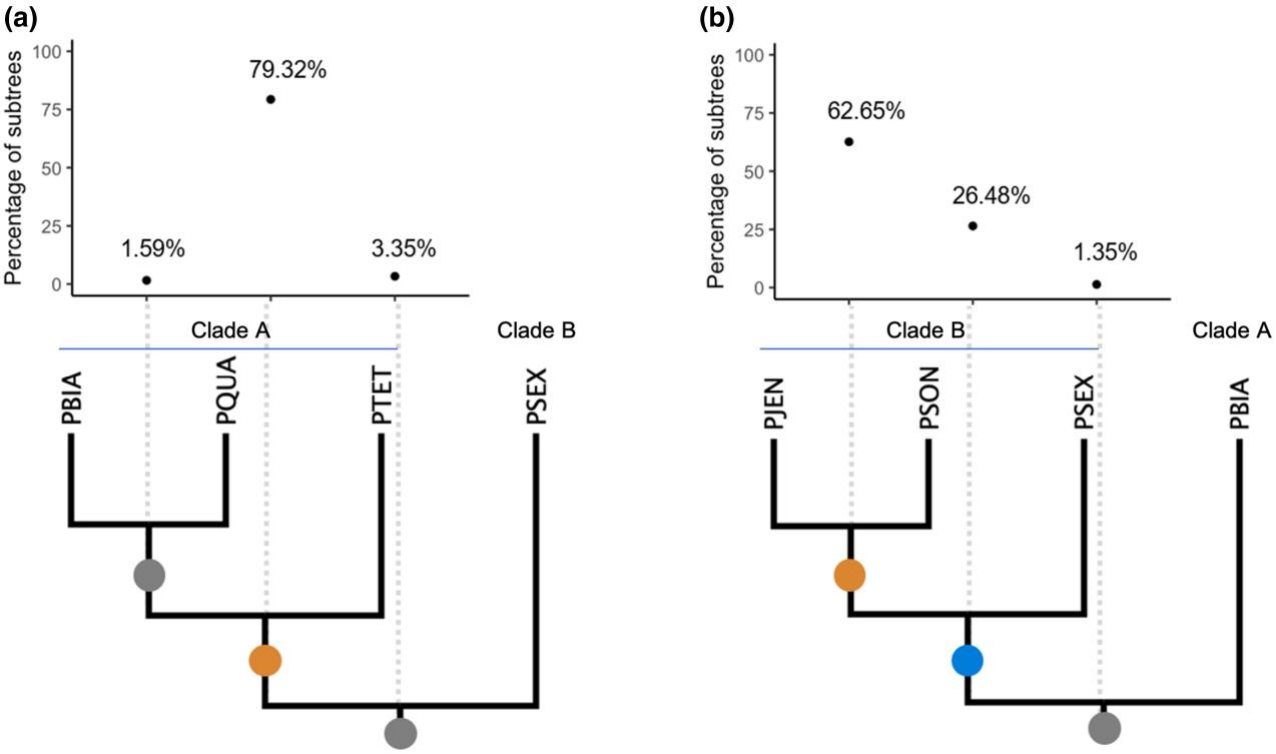
MAPS (Li et al. 2015) inference of the placement of WGD events along the branches of two different subtree topologies. The first subtree (a) contains three species from clade A and one species from clade B. The second subtree (b) contains three species from clade B and one species from clade A. The dot plot shows the percentage of gene trees supporting different WGD(s) placement. The circles on the tree branches are colored based on the percentage of subtree support of WGD(s) placement. Orange, ≥50%; blue, <50% and ≥25%; and grey, <25%.

For each of the 19,802 orthologous gene families identified with PoFF (Lechner et al. 2014), a maximum likelihood gene tree was built using IQ-TREE (Nguyen et al. 2015). In the 19,802 gene trees, we noticed that many formed two distinct clusters, one containing gene copies from *P. sexaurelia*, *P. jenningsi*, and *P. sonneborni*, and the other cluster formed by gene copies from the rest of the *P. aurelia* species. As there has been a previous suggestion of such division of the *P. aurelia* complex (Sellis et al. 2021), we divided the 13 *P. aurelia* species into 2 subclades (Sellis et al. 2021): clade A (*P. primaurelia*, *P. biaurelia*, *P. tetraurelia*, *P. pentaurelia*, *Paramecium octaurelia*, *Paramecium novaurelia*, *P. decaurelia*, *P. dodecaurelia*, *P. tredecaurelia*, and *Paramecium quadecaurelia*) and clade B (*P. sexaurelia*, *P. jenningsi*, and *P. sonneborni*). We then evaluated the hypothesis that the gene tree topologies could be explained by an alternative evolutionary history of the *P. aurelia* complex in which two independent WGD events occurred, each in a different subclade (supplementary fig. S3, Supplementary Material online), as opposed to the conventional view in which the entire *P. aurelia* clade originates after the most recent WGD. To test this hypothesis, we used two ladderized subtrees from each gene tree and ran the multi-taxon paleopolyploid search algorithm (MAPS) to estimate the percentage of gene trees that support different WGD placements (Li et al. 2015). When sampling three taxa from clade A and using one species from clade B as out group, the majority of subtrees support the placement of one WGD event at the base of clade B (fig. 4*a*). However, when including three taxa from clade B and one from clade A (fig. 4*b*), MAPS yielded different results and only 26% of subgene trees supported the placement of WGD event on the split between clades A and B. A total of 63% of subgene trees supported an independent duplication shared by *P. jenningsi* and *P. sonneborni*, suggesting distinct gene loss patterns in different *Paramecium* species. In both scenarios, only 1–3% of gene trees support the WGD placement on the root branch, indicating that the gene retention patterns might be different between the two subclades. Thus, we cannot rule out the possibility of a scenario of two separate WGDs, although there is also no compelling evidence for rejecting a one-WGD model.

### Patterns of Gene Conversions in Two Subclades

If gene conversion occurs, a gene pair would appear younger than real paralogous pairs created by WGD (Wang et al. 2011). Previous studies have shown that gene conversions are common in *Paramecium* paralogous pairs after WGD (McGrath, Gout, Doak, et al. 2014; McGrath, Gout, Johri, et al. 2014). Thus, we plotted the *K*_s_ values along each paralogon within each species (Materials and Methods) and used a change point detection method to find sudden downward or upward shifts in *K*_s_ values (Killick and Eckley 2014). *Paramecium sonneborni* contains the highest number of paralogons exhibiting significant shifts in *K*_s_ (9 out of 104; supplementary table S5 and fig. S4, Supplementary Material online). Interestingly, *P. primaurelia* and *P. tredecaurelia* also have multiple paralogons with change points in *K*_s_ (supplementary table S5, Supplementary Material online), indicating frequent occurrence of gene conversion in post-WGD *Paramecium* species, regardless of their subclades (McGrath, Gout, Doak, et al. 2014; McGrath, Gout, Johri, et al. 2014).

### Interplay with Single-Gene Duplications

Despite the very large numbers of genes within *P. aurelia* species resulting from WGDs, it has been noted that additional single-gene duplications are rare (Aury et al. 2006; McGrath, Gout, Doak, et al. 2014). We searched for evidence of recent single-gene duplication in all *P. aurelia* genomes and confirmed their extreme paucity, with a median of just 28 recent single-gene duplications per genome (supplementary table S4, Supplementary Material online; Materials and Methods). This number contrasts with the thousands of gene losses that have happened since the most recent *Paramecium* WGD in each of these lineages and is in very sharp contrast to the recurrent single-gene duplications observed in all other eukaryotes (Lynch 2007; Gao and Lynch 2009). Despite the small number of recent single-gene duplications, we were able to detect a bias for these duplications toward genes that have already lost their ohnologs from the most recent *Paramecium* WGD. Genes that had reverted to single-copy status since the WGD are on average twice as likely to be part of a subsequent recent single-gene duplication as those that had maintained both WGD–derived duplicates (supplementary table S4, Supplementary Material online). We interpret these observations as additional evidence in support of dosage sensitivity playing a major role in gene retention and duplication in *Paramecium*. The genes that have had a copy lost following the recent WGD are also more permissive to subsequent single-gene duplications, suggesting that dosage-induced constraints are stronger on the retained duplicates relative to single-copy genes and that perturbations of established post-WGD dosage balance are at least slightly detrimental, which could be the reason why both copies of these genes were retained in the first place (Birchler and Veitia 2012).

## Discussion

This view of postduplication genome evolution in 13 *Paramecium* species sharing a common WGD represents the most fine-scaled analysis of the historical demography of duplicated genes performed in any lineage of eukaryotes. All species have undergone substantial gene loss since the WGD, to the point that 40–60% of paralogs created by the WGD (ohnologs) have lost one copy. Despite this significant variation in retention rate between species, we observed a number of strikingly similar trends in gene retention and loss across all 13 *P. aurelia* species. Most notably, highly expressed genes are systematically overretained in two copies. Different functional categories of genes also showed consistent patterns of over- and underretention across the entire phylogeny of *P. aurelia*. The observation that both expression level and functional category influence the probability of post-WGD retention in a way that is consistent across many species indicates that the fate of ohnologs is in part predetermined (Hao et al. 2018, 2022). Although we cannot exclude the possibility that the number of mutational targets for neo- and subfunctionalization depends on the expression level and functional category in the cryptic *P. aurelia* species complex, the patterns observed here are at odds with random mutations, creating new functions as the main force driving post-WGD gene retention. It should also be noted that, with the potential exception of genes lost very early following the genome duplication, purifying selection has been operating to maintain duplicated copies for some time before allowing gene loss. We observe an average *K*_a_/*K*_s_ between ohnologs in *P. aurelia* species of just 0.05, indicating strong purifying selection against pseudogenization operating since the WGD (Johri et al. 2022).

In an effort to test whether independent or shared WGD(s) had occurred in two *P. aurelia* subclades, we found that the WGD(s) placement using a gene tree method would yield different results when inferred from different taxon samplings across two subclades of the *P. aurelia* species, suggesting that the gene loss and retention pattern might be different for species within subclades A and B. However, no significant difference in paralogous *K*_s_ distributions was observed (supplementary fig. S5, Supplementary Material online). Thus, even if the two subclades experienced independent WGD events, these events must have occurred at around the same time.

We also note that retained duplicates not only exhibit higher expression levels but are also less likely to experience later single-gene duplications. Together, the evidence indicates that gene dosage balance plays an important role in determining the loss/retention fate of WGD–derived ohnologs (Birchler and Veitia 2012). The relatively high retention rate in *Paramecium* when compared with other post-WGD eukaryotic species in concert with the scarcity of single-gene duplications in all *Paramecium* genomes studied here again supports the view that dosage constraints are the major drivers for post-WGD genome evolution.

Finally, we hope that this data set, along with other efforts of *Paramecium* genome assemblies (Sellis et al. 2021), will be useful to other researchers studying WGDs while also helping establish *Paramecium* as a model species for studying WGDs, alongside yeast.

## Materials and Methods

### Genome Sequencing, Assembly, and Annotation

*Paramecium* cells that had recently undergone autogamy (a self-fertilization process that creates 100% homozygous individuals) were grown in up to 2 l of Wheat Grass Powder medium (Pines International) before being starved and harvested. *Paramecium* cells were separated from the remaining food bacteria by filtration on a 10 *µ*m Nitex membrane. Macronuclei were isolated away from other cellular debris by gentle lysis of the cell membrane and sucrose density separation. DNA was extracted and purified using a cetyltrimethyl ammonium bromide (CTAB) protocol (Doyle and Doyle 1987). DNA libraries were constructed with the Illumina Nextera DNA library preparation kit following manufacturer's recommendations, and sequencing was performed on a HiSeq 2500 machine producing 2 × 150 nt reads. Reads were trimmed for adapter sequences and quality (3′ end trimming down to *Q* = 20) with cutadapt version 1.15 (Martin 2011). Genome assembly was performed with SPades version 3.11 (Nurk et al. 2013) with default parameters. Final assembly was cleaned up by removing short scaffolds (less than 1 kb) and scaffolds with strong Blast hits to bacterial genomes. Genome annotation was done with the EuGene pipeline (Foissac et al. 2008) using the RNA-seq data (see below) generated for each data as described in Arnaiz et al. (2017). The list of *Paramecium* strains used in this study is as follows: *P. primaurelia* Ir4-2, *P. biaurelia* V1–4 (McGrath, Gout, Johri, et al. 2014), *P. tetraurelia* 51 (Aury et al. 2006; Arnaiz et al. 2012), *P. pentaurelia* 87 (Sellis et al. 2021), *P. sexaurelia* AZ8-4, *P. octaurelia* K8, *P. novaurelia* TE, *P. decaurelia* 223, *P. dodecaurelia* 274, *P. tredecaurelia* d13-2 (derivative of 209), *P. quadecaurelia* N1A, *P. jenningsi* M, *P. sonneborni* ATCC30995 (Sellis et al. 2021), *P. multimicronucleatum* MO 3c4, and *P. caudatum* 43c3d (McGrath, Gout, Doak, et al. 2014). Sellis et al. (Sellis et al. 2021) also reported four MAC genome assemblies, including two different strains *P. octaurelia* 138 and *P. primaurelia* AZ9-3, and an improved version of the *P. sonneborni* ATCC30995 assembly after scaffolding and gap-closing, allowing further research to compare genomic diversity within different populations.

### RNA-seq and Expression-Level Quantification

*Paramecium* cells were grown in ∼1 l of Wheat Grass Powder medium to midlog phase before harvesting. Cells were purified away from bacteria by filtration on a 10 *µ*m Nitex membrane. Whole-cell RNA was isolated using TRIzol (Ambion) and the manufacturer's suggested protocol for tissue culture cells. cDNA libraries were prepared with the Illumina TruSeq library preparation kit following the manufacturer's suggested protocol and then sequenced with Illumina single-end 150 nt reads. RNA-seq reads were mapped to each corresponding genome with Bowtie/TopHat (Langmead et al. 2009; Kim et al. 2013), and transcript abundance (FPKM) was computed using cufflinks (Trapnell et al. 2010) with –multi-read-correct and –frag-bias-correct options to obtain values of FPKM for each predicted protein-coding gene. Expression level was defined for each gene as the log(FPKM + 0.1), the small offset (0.1) being added to include genes with FPKM values of zero even after log-transformation. Based on the expression level in *P. caudatum*, the genes were binned into 14 subgroups, with about 1,322 genes per group. Within each bin of the *P. caudatum* genes, the average percentage of gene retention was then calculated for all *P. aurelia* genes that are orthologous to these *P. caudatum* genes. The average *P. aurelia* gene retention rate was compared with average *P. caudatum* gene expression fold change.

### WGD Paralogon Inference

Paralogs in the 13 *Paramecium* genomes that were derived from the 3 successive WGDs were annotated using the pipeline initially described in Aury et al. (2006). Briefly, reciprocal best hits (RBH) of protein-coding genes were found using global all-against-all Blast, scaffolds were scanned, and windows with RBHs were merged into paralogous blocks, which are large blocks of synteny derived from the most recent WGD. These paralogous blocks were then extended by adding non-RBH syntenic matches and then fused into paralogons. Retained and lost duplicates were identified within these blocks. Ancestral (pre-WGD) genome reconstruction is then performed by fusion of the paralogous blocks with the following criteria: If both paralogs are still retained, one copy is randomly chosen to be incorporated in the ancestral genome, and if one copy has been lost, the remaining copy is included at the ancestral locus. The process is then repeated with the ancestrally reconstructed genome for more ancient genome duplications. These ancestral paralogon blocks were included in supplementary Data S1, Supplementary Material online, and provided the gene order information used in later analyses.

### Orthology Relationship Inference

Protein-coding genes from 13 *P. aurelia* genomes were grouped into 19,802 gene families using the orthology detection tool PoFF (Lechner et al. 2014). Genes in every gene family were aligned using MUSCLE (Edgar 2004b), and a maximum likelihood gene tree was built using IQ-TREE (Nguyen et al. 2015). Orthologs were first assigned using a combination of PoFF (Lechner et al. 2014) and in-house scripts.

For our initial round of orthology prediction, we used PoFF across all 13 *P. aurelia* species. Following this first round, an “orthology score” was attributed to each pair of scaffolds linked by at least one orthologous gene pair. The score was defined as the number of genes being annotated as orthologous between the two scaffolds by PoFF. Orthology relationships were then updated with the following criteria: 1-to-2 orthology relationships where the “2” corresponds to two WGD–derived paralogs were converted to 1-to-1, selecting the gene on the scaffold with the highest orthologous score as being the ortholog. Orthology relationship with *P. caudatum* and *P. multimicronucleatum* was then inferred by selecting the genes in these two species with the highest Blast hit scores to the entire *P. aurelia* orthologs family.

### Resolving the Orthology Relations between Paralogons in Different Species

Next, we try to resolve the orthologous relationships across paralogons in 13 *P. aurelia* species. The 19,802 gene trees were rerooted at the split between clade A and clade B using Python library DendroPy version 4.4.0 (Sukumaran and Holder 2010). Starting from all the sister gene pairs in the tree, orthologous gene groups were extended step by step, until no further genes could be added to the group. Thus, orthologous gene groups with various sizes were inferred from all the gene trees. Then, a paralogon graph was built using Python package NetworkX version 2.5 (Hagberg et al. 2008). In this network, the nodes represent the paralogons. Two paralogons are connected if the genes residing on these paralogons belong to the same orthologous group. The edge weights are the number of gene trees supporting the connection. Starting from the thickest edge, each path of paralogons that are connected by the greatest number of gene tree supports was retrieved, resulting in the most possible orthologous relations between paralogons in 13 species. The orthologous blocks were included as supplementary Data S2, Supplementary Material online; the number after each species name represents paralogon identifier.

### Building the Phylogenetic Tree

Protein sequences for orthologous genes that were present in a single copy in at least half of the *P. aurelia* species were aligned to their corresponding orthologous sequences from *P. caudatum* and *P. multimicronucleatum*, using MUSCLE version 3.8 (Edgar 2004a). Alignments were cleaned using gblocks (Castresana 2000), and a phylogenetic tree was build using the distance method implemented in SeaView (Gouy et al. 2010).

### Inferring Loss of Gene Duplicate

Branch-specific loss of gene duplicates were inferred by parsimony using ancestral reconstruction with in-house scripts. We assumed that probability of gain of duplicates is zero. Missing data were encoded as “NA” such that ancestor (child1 = “NA” and child2 = “gene duplicate present”) = “gene duplicate present”; ancestor (child1 = “NA” and child2 = “only one duplicate present”) = “NA”; and ancestor (child1 = “NA” and child2 = “NA”) = “NA”. In total, 9983 gene duplicate pairs were present in the ancestor (or root) of all *P. aurelia* species. Probability of survival was obtained for every node in the phylogenetic tree (based on protein sequences) as 1.0 − (number of duplicates present in the root − number of duplicates present at the node)/number of duplicates present in the root.

### Inferring the Placement of WGD Event(s)

From each of the 19,802 gene trees, two laddered subtrees were parsed. The first tree consists of *P. biaurelia*, *P. quadecaurelia*, and *P. tetraurelia* from subclade A and *P. sexaurelia* from subclade B. The second tree consists of *P. jenningsi*, *P. sonneborni*, and *P. sexaurelia* from subclade B and *P. biaurelia* from subclade A. These subtrees were then filtered to make sure that there is at least one gene copy representing each taxon. Using the multi-taxon paleopolyploid search algorithm (MAPS; Li et al. 2015), we estimated the percentage of subtrees supporting the placement of the WGD events on different branches.

### Identifying the Gene Conversion Patterns within the Two *P. aurelia* Clades

The synonymous substitution rate *K*_s_ between homoeologs was calculated using codeml under F1 × 4 model (Yang 2007). The *K*_s_ values along each paralogon were plotted against the gene order. If gene conversion occurred between a pair of genes created by the WGD, this pair of genes would look more similar than the other homoeologous pairs (McGrath, Gout, Doak, et al. 2014). If the conversion spanned across longer regions with multiple genes, we would observe lower *K*_s_ valleys along the *K*_s_ plots of these paralogons. We then employed change point detection method to detect the unexpectedly younger homologous strata using the package changepoint version 2.2.2 in R 4.0.2 (Killick and Eckley 2014).

### Finding Single-Gene Duplications

We started the search for recent single-gene duplications in each species with a Blast (Altschul et al. 1990) search of a database containing all protein-coding genes against itself. After removing self-hits, we selected pairs of reciprocal best Blast hits and removed the pairs that were already annotated as being WGD–derived paralogs. We then removed hits that were not inside a paralogon (a block of WGD–related genes with preserved synteny) to avoid the possibility of “contamination” with WGD–related paralogs that would have been missed by the initial annotation because of subsequent gene relocation. Finally, we computed the rate of synonymous substitution for each remaining pair of genes and retained only those with a synonymous substitution below 1.0. *Paramecium sonneborni* was excluded from this analysis because of the presence of micronucleus-derived sequences in the genome assembly.

## Supplementary Material

msad107_Supplementary_Data

## Data Availability

The HiSeq 2500 2 × 150 bp raw reads were deposited to Sequence Read Archive (SRA) with BioSample accessions SAMN28886867, SAMN28886868, SAMN28886869, SAMN28886870, SAMN28886871, SAMN28886872, and SAMN28886873. The RNA-seq Illumina single-end 150 bp raw reads were deposited to SRA under BioProject ID PRJNA849663. The annotated genomes were uploaded to the *Paramecium*DB (https://Paramecium.i2bc.paris-saclay.fr).
