# Supplementary material for: Dynamics of Gene Loss following Ancient Whole-Genome Duplication in the Cryptic Paramecium Complex
Source: Mol Biol Evol. 2023 May 8;40(5):msad107. doi: 10.1093/molbev/msad107 (PMC10195154; doi:10.1093/molbev/msad107)
Supplement: msad107_Supplementary_Data [file msad107_supplementary_data.docx]

## **Table S1.** Assembly statistics and average post-recent-WGD retention rate in all *Paramecium* species.

| Species | Assembly size (Mbp) | # Scaffolds | Largest scaffold (Mbp) | N50 (Mbp) | # Protein-coding genes | % WGD1 paralogs retained |
| --- | --- | --- | --- | --- | --- | --- |
| *P. biaurelia* V1-4 | 76.98 | 2362 | 1.048449 | 0.150218 | 40261 | 54.07 |
| *P. decaurelia* 223 | 71.91 | 1076 | 0.697401 | 0.189418 | 40810 | 52.08 |
| *P. dodecaurelia* 274 | 71.63 | 1147 | 0.653349 | 0.176048 | 41085 | 51.44 |
| *P. jenningsi* M | 65.35 | 984 | 0.807764 | 0.212635 | 37098 | 58.3 |
| *P. novaurelia* TE | 64.79 | 1877 | 0.624896 | 0.078722 | 35534 | 54.87 |
| *P. octaurelia* K8 | 72.98 | 585 | 1.072884 | 0.448897 | 38668 | 50.98 |
| *P. pentaurelia* 87 | 87.29 | 310 | 1.263468 | 0.515355 | 41676 | 55.03 |
| *P. primaurelia* Ir4-2 | 71.02 | 677 | 0.994169 | 0.469549 | 34474 | 52.48 |
| *P. quadecaurelia* N1A | 59.12 | 710 | 0.743565 | 0.223945 | 33793 | 42.41 |
| *P. sexaurelia* AZ8-4 | 68.02 | 547 | 1.303432 | 0.425007 | 36094 | 43.03 |
| *P. sonneborni* ATCC30995 | 98.06 | 572 | 1.141545 | 0.500206 | 49951 | 56.43 |
| *P. tetraurelia* 51 | 72.10 | 697 | 0.98076 | 0.413026 | 40460 | 50.71 |
| *P. tredecaurelia* d13-2 | 65.93 | 245 | 1.22386 | 0.496921 | 36179 | 39.00 |

## **Table S2**. Average retention rate for different functional categories. For each functional category with at least 20 genes in *P. caudatum*, the post-WGD retention rate was computed across all *P. aurelia* species (column #3). We then randomly draw the same number of genes from the rest of the genome (*i.e.*, genes that are not in this functional category) with similar expression levels as the genes from the functional category considered. We then compute the average retention for these randomly drawn genes and repeat the random drawing 100 times to obtain an average retention rate after correction for expression level (column #4). Column #5 is the standard deviation of the retention rate across 100 random drawings.

| Gene Ontology ID | Name | Number of *P. caudatum* genes in this functional category | Average retention rate | Average retention rate after correction for expression level | standard deviation |
| --- | --- | --- | --- | --- | --- |
| GO:0007274 | neuromuscular synaptic transmission | 21 | 0.234042553 | 0.489689626 | 0.146531315 |
| GO:0016799 | hydrolase activity, hydrolyzing N-glycosyl compounds | 25 | 0.276470588 | 0.555562415 | 0.096006509 |
| GO:0006858 | extracellular transport | 65 | 0.369047619 | 0.482291384 | 0.069070174 |
| GO:0006486 | protein glycosylation | 119 | 0.374277457 | 0.564433859 | 0.052919056 |
| GO:0042626 | ATPase-coupled transmembrane transporter activity | 69 | 0.388059701 | 0.539896634 | 0.068484285 |
| GO:0016779 | nucleotidyltransferase activity | 56 | 0.411764706 | 0.581520186 | 0.059600064 |
| GO:0006399 | tRNA metabolic process | 46 | 0.420054201 | 0.60267722 | 0.081979525 |
| GO:0016836 | hydro-lyase activity | 39 | 0.422712934 | 0.64706596 | 0.075342789 |
| GO:0004620 | phospholipase activity | 22 | 0.435028249 | 0.655461394 | 0.110010149 |
| GO:0019221 | cytokine-mediated signaling pathway | 31 | 0.436781609 | 0.526031346 | 0.100265484 |
| GO:0007169 | transmembrane receptor protein tyrosine kinase signaling pathway | 30 | 0.438297872 | 0.533760684 | 0.083166468 |
| GO:0016854 | racemase and epimerase activity | 41 | 0.453703704 | 0.680423558 | 0.065406964 |
| GO:0003899 | DNA-directed 5'-3' RNA polymerase activity | 32 | 0.453815261 | 0.599618321 | 0.081966455 |
| GO:0006778 | porphyrin-containing compound metabolic process | 21 | 0.453947368 | 0.650411281 | 0.10376394 |
| GO:0009636 | response to toxic substance | 80 | 0.463035019 | 0.592689931 | 0.054312713 |
| GO:0006732 | obsolete coenzyme metabolic process | 76 | 0.469448584 | 0.658887558 | 0.047713445 |
| GO:0004190 | aspartic-type endopeptidase activity | 33 | 0.472140762 | 0.582711443 | 0.077145785 |
| GO:0006633 | fatty acid biosynthetic process | 23 | 0.475 | 0.642629717 | 0.099765479 |
| GO:0008237 | metallopeptidase activity | 69 | 0.477011494 | 0.619098633 | 0.063174934 |
| GO:0008643 | carbohydrate transport | 58 | 0.479757085 | 0.542718447 | 0.061275713 |
| GO:0006631 | fatty acid metabolic process | 91 | 0.481804949 | 0.661268939 | 0.045590289 |
| GO:0008202 | steroid metabolic process | 122 | 0.485148515 | 0.610632782 | 0.04114099 |
| GO:0004521 | endoribonuclease activity | 40 | 0.488721805 | 0.599632353 | 0.077823547 |
| GO:0016298 | lipase activity | 31 | 0.489082969 | 0.622817991 | 0.082156109 |
| GO:0006635 | fatty acid beta-oxidation | 29 | 0.492307692 | 0.73191848 | 0.071318879 |
| GO:0043574 | peroxisomal transport | 41 | 0.495670996 | 0.56825512 | 0.061771251 |
| GO:0004383 | guanylate cyclase activity | 25 | 0.497777778 | 0.475339155 | 0.078290712 |
| GO:0008234 | cysteine-type peptidase activity | 130 | 0.502819549 | 0.614184476 | 0.038226742 |
| GO:0003779 | actin binding | 39 | 0.505263158 | 0.604309285 | 0.078582758 |
| GO:0016757 | glycosyltransferase activity | 147 | 0.505543237 | 0.576523389 | 0.043393465 |
| GO:0003724 | RNA helicase activity | 65 | 0.506896552 | 0.572213978 | 0.058812837 |
| GO:0016831 | carboxy-lyase activity | 27 | 0.510067114 | 0.675515233 | 0.114128595 |
| GO:0005245 | voltage-gated calcium channel activity | 31 | 0.518691589 | 0.436756461 | 0.099568941 |
| GO:0005248 | voltage-gated sodium channel activity | 31 | 0.518691589 | 0.439318958 | 0.102508875 |
| GO:0019227 | neuronal action potential propagation | 31 | 0.518691589 | 0.456333373 | 0.091858368 |
| GO:0004016 | adenylate cyclase activity | 37 | 0.519637462 | 0.50148415 | 0.067961118 |
| GO:0006865 | amino acid transport | 28 | 0.520408163 | 0.566520368 | 0.073991343 |
| GO:0008203 | cholesterol metabolic process | 78 | 0.52265861 | 0.590909091 | 0.053399985 |
| GO:0008233 | peptidase activity | 348 | 0.523140213 | 0.619757664 | 0.024601674 |
| GO:0008415 | acyltransferase activity | 81 | 0.523972603 | 0.632519077 | 0.060206648 |
| GO:0051179 | localization | 161 | 0.526264591 | 0.58872066 | 0.045959298 |
| GO:0005216 | ion channel activity | 291 | 0.528110599 | 0.506636487 | 0.026677846 |
| GO:0006403 | RNA localization | 156 | 0.529292929 | 0.582263729 | 0.045357989 |
| GO:0005244 | voltage-gated ion channel activity | 205 | 0.529553679 | 0.483209142 | 0.03128873 |
| GO:0005261 | cation channel activity | 205 | 0.529553679 | 0.481737311 | 0.030961332 |
| GO:0016853 | isomerase activity | 150 | 0.530883503 | 0.658453609 | 0.034698861 |
| GO:0022857 | transmembrane transporter activity | 677 | 0.531031881 | 0.549919615 | 0.01813288 |
| GO:0005249 | voltage-gated potassium channel activity | 174 | 0.531163435 | 0.486572911 | 0.034521274 |
| GO:0015276 | ligand-gated ion channel activity | 183 | 0.531628533 | 0.501379906 | 0.035526436 |
| GO:0016829 | lyase activity | 136 | 0.531858407 | 0.618611173 | 0.032537289 |
| GO:0005215 | transporter activity | 685 | 0.532465116 | 0.550289449 | 0.019074052 |
| GO:0004812 | aminoacyl-tRNA ligase activity | 26 | 0.532967033 | 0.660018295 | 0.090349375 |
| GO:0006796 | phosphate-containing compound metabolic process | 393 | 0.533524904 | 0.574047245 | 0.02195181 |
| GO:0015171 | amino acid transmembrane transporter activity | 74 | 0.536776213 | 0.61199441 | 0.052106555 |
| GO:0043855 | cyclic nucleotide-gated monoatomic ion channel activity | 158 | 0.537827715 | 0.4820376 | 0.04044217 |
| GO:0006508 | proteolysis | 645 | 0.539671065 | 0.611549099 | 0.017638788 |
| GO:0007292 | female gamete generation | 22 | 0.542372881 | 0.618230519 | 0.115993382 |
| GO:0007269 | neurotransmitter secretion | 137 | 0.542801556 | 0.531304038 | 0.042007092 |
| GO:0008236 | serine-type peptidase activity | 71 | 0.543010753 | 0.618707037 | 0.049347562 |
| GO:0016866 | intramolecular transferase activity | 21 | 0.543046358 | 0.667572599 | 0.103376632 |
| GO:0006874 | cellular calcium ion homeostasis | 62 | 0.543429844 | 0.538189099 | 0.065068905 |
| GO:0008324 | cation transmembrane transporter activity | 345 | 0.545021962 | 0.522376111 | 0.024849128 |
| GO:0006898 | receptor-mediated endocytosis | 40 | 0.548295455 | 0.618387968 | 0.060184976 |
| GO:0004872 | signaling receptor activity | 300 | 0.548682703 | 0.532608597 | 0.027427164 |
| GO:0032568 | transcription by RNA polymerase II | 27 | 0.548717949 | 0.634531633 | 0.096569335 |
| GO:0006629 | lipid metabolic process | 528 | 0.549334557 | 0.615724668 | 0.019473355 |
| GO:0007601 | visual perception | 91 | 0.553524804 | 0.577415744 | 0.043486918 |
| GO:0006766 | vitamin metabolic process | 48 | 0.556430446 | 0.652849741 | 0.070247083 |
| GO:0006869 | lipid transport | 158 | 0.557198444 | 0.596669665 | 0.036893674 |
| GO:0016787 | hydrolase activity | 1487 | 0.558446889 | 0.597286431 | 0.012039042 |
| GO:0006310 | DNA recombination | 36 | 0.558823529 | 0.543663358 | 0.074226274 |
| GO:0016874 | ligase activity | 472 | 0.559277738 | 0.591887846 | 0.021821233 |
| GO:0009110 | vitamin biosynthetic process | 40 | 0.561128527 | 0.678744995 | 0.069059856 |
| GO:0008168 | methyltransferase activity | 87 | 0.561983471 | 0.594578527 | 0.042082156 |
| GO:0004518 | nuclease activity | 115 | 0.562439497 | 0.596790342 | 0.041963497 |
| GO:0042592 | homeostatic process | 65 | 0.5639413 | 0.554342432 | 0.065854307 |
| GO:0016407 | acetyltransferase activity | 49 | 0.564516129 | 0.639362059 | 0.060295687 |
| GO:0006418 | tRNA aminoacylation for protein translation | 31 | 0.565957447 | 0.628814464 | 0.101693925 |
| GO:0009566 | fertilization | 32 | 0.56684492 | 0.536934576 | 0.083614356 |
| GO:0007249 | I-kappaB kinase/NF-kappaB signaling | 32 | 0.567307692 | 0.593999698 | 0.104045253 |
| GO:0019882 | antigen processing and presentation | 38 | 0.567867036 | 0.581906808 | 0.070452149 |
| GO:0006917 | apoptotic process | 105 | 0.569174757 | 0.561401326 | 0.045071878 |
| GO:0006811 | monoatomic ion transport | 549 | 0.569762123 | 0.554337419 | 0.020269084 |
| GO:0006968 | cellular defense response | 71 | 0.570605187 | 0.596593722 | 0.051513815 |
| GO:0019207 | kinase regulator activity | 354 | 0.570843291 | 0.564871232 | 0.02617709 |
| GO:0016491 | oxidoreductase activity | 373 | 0.571193686 | 0.671011286 | 0.020019427 |
| GO:0003924 | GTPase activity | 114 | 0.571862348 | 0.595268636 | 0.04031664 |
| GO:0006812 | cation transport | 534 | 0.573735955 | 0.553777185 | 0.017962939 |
| GO:0004842 | ubiquitin-protein transferase activity | 253 | 0.574539363 | 0.57580668 | 0.028781897 |
| GO:0004386 | helicase activity | 105 | 0.574795082 | 0.575130825 | 0.041579722 |
| GO:0007267 | cell-cell signaling | 314 | 0.575314657 | 0.548079255 | 0.025795913 |
| GO:0007268 | chemical synaptic transmission | 306 | 0.577429984 | 0.53979004 | 0.027135871 |
| GO:0003746 | translation elongation factor activity | 44 | 0.578313253 | 0.666666667 | 0.070961136 |
| GO:0006790 | sulfur compound metabolic process | 36 | 0.579268293 | 0.638068311 | 0.068632449 |
| GO:0019210 | kinase inhibitor activity | 187 | 0.58137931 | 0.590843215 | 0.035479906 |
| GO:0006807 | nitrogen compound metabolic process | 77 | 0.584664537 | 0.649446961 | 0.050325494 |
| GO:0006644 | phospholipid metabolic process | 178 | 0.585334199 | 0.585929365 | 0.037355682 |
| GO:0003824 | catalytic activity | 4369 | 0.585671807 | 0.5935817 | 0.005863404 |
| GO:0009187 | cyclic nucleotide metabolic process | 60 | 0.586715867 | 0.539175111 | 0.06083267 |
| GO:0006401 | RNA catabolic process | 63 | 0.590032154 | 0.604843074 | 0.053107448 |
| GO:0006457 | protein folding | 145 | 0.594240838 | 0.653879922 | 0.03628301 |
| GO:0006810 | transport | 1611 | 0.59431988 | 0.568200769 | 0.010149306 |
| GO:0007166 | cell surface receptor signaling pathway | 443 | 0.596117552 | 0.572894671 | 0.019908474 |
| GO:0007186 | G protein-coupled receptor signaling pathway | 295 | 0.597446489 | 0.564506516 | 0.023841852 |
| GO:0005484 | SNAP receptor activity | 35 | 0.597701149 | 0.608429459 | 0.080512877 |
| GO:0031201 | SNARE complex | 35 | 0.597701149 | 0.602647715 | 0.087591898 |
| GO:0051169 | nuclear transport | 74 | 0.597765363 | 0.625621272 | 0.05914454 |
| GO:0002504 | antigen processing and presentation of peptide or polysaccharide antigen via MHC class II | 31 | 0.597785978 | 0.527007907 | 0.08453823 |
| GO:0006817 | phosphate ion transport | 63 | 0.598058252 | 0.619281678 | 0.056993188 |
| GO:0007155 | cell adhesion | 52 | 0.598108747 | 0.581744745 | 0.064756165 |
| GO:0008152 | metabolic process | 5215 | 0.600642628 | 0.595770711 | 0.005223187 |
| GO:0003743 | translation initiation factor activity | 96 | 0.602739726 | 0.64547651 | 0.04478873 |
| GO:0006519 | amino acid metabolic process | 330 | 0.604129583 | 0.61825514 | 0.025377792 |
| GO:0006520 | cellular amino acid metabolic process | 330 | 0.604129583 | 0.616299452 | 0.024928958 |
| GO:0044238 | primary metabolic process | 5024 | 0.604410336 | 0.593308509 | 0.004944078 |
| GO:0006464 | cellular protein modification process | 1738 | 0.604983459 | 0.564844082 | 0.011489657 |
| GO:0006281 | DNA repair | 194 | 0.605921388 | 0.550584117 | 0.030666435 |
| GO:0007600 | sensory perception | 206 | 0.606472492 | 0.543615465 | 0.03394977 |
| GO:0003712 | transcription coregulator activity | 162 | 0.606725146 | 0.563643865 | 0.035959942 |
| GO:0006887 | exocytosis | 138 | 0.607808341 | 0.55866375 | 0.034633678 |
| GO:0050877 | nervous system process | 630 | 0.607819669 | 0.554466472 | 0.016475023 |
| GO:0009063 | amino acid catabolic process | 64 | 0.607936508 | 0.672296024 | 0.043966377 |
| GO:0022904 | respiratory electron transport chain | 132 | 0.610287707 | 0.668519305 | 0.035544637 |
| GO:0016788 | hydrolase activity, acting on ester bonds | 585 | 0.611342786 | 0.577465828 | 0.02002226 |
| GO:0016740 | transferase activity | 1928 | 0.61338623 | 0.567296534 | 0.010076513 |
| GO:0043234 | protein-containing complex | 66 | 0.613537118 | 0.588489516 | 0.063894711 |
| GO:0005975 | carbohydrate metabolic process | 498 | 0.615015591 | 0.612259526 | 0.021085555 |
| GO:0019538 | protein metabolic process | 2943 | 0.615181213 | 0.590044959 | 0.007519785 |
| GO:0043226 | organelle | 72 | 0.616260163 | 0.635805719 | 0.049196672 |
| GO:0007154 | cell communication | 2296 | 0.616783363 | 0.554679521 | 0.008853733 |
| GO:0003008 | system process | 712 | 0.6167915 | 0.549273085 | 0.015039702 |
| GO:0008092 | cytoskeletal protein binding | 260 | 0.617073171 | 0.565002659 | 0.029606431 |
| GO:0009950 | dorsal/ventral axis specification | 47 | 0.617210682 | 0.663410984 | 0.062360064 |
| GO:0006260 | DNA replication | 145 | 0.618650493 | 0.581797669 | 0.041344786 |
| GO:0007165 | signal transduction | 2188 | 0.622179628 | 0.558366312 | 0.009927466 |
| GO:0015931 | nucleobase-containing compound transport | 83 | 0.622252747 | 0.605669153 | 0.048499006 |
| GO:0008081 | phosphoric diester hydrolase activity | 37 | 0.622807018 | 0.568220644 | 0.072849833 |
| GO:0008652 | cellular amino acid biosynthetic process | 203 | 0.623033354 | 0.605490127 | 0.030499566 |
| GO:0003887 | DNA-directed DNA polymerase activity | 28 | 0.623616236 | 0.579070837 | 0.078586346 |
| GO:0007399 | nervous system development | 557 | 0.62479945 | 0.569958372 | 0.019260716 |
| GO:0045182 | translation regulator activity | 122 | 0.625137326 | 0.649301998 | 0.043009541 |
| GO:0005515 | protein binding | 1428 | 0.625382263 | 0.57523976 | 0.011315686 |
| GO:0031202 | RNA splicing, via transesterification reactions | 278 | 0.625566636 | 0.609918846 | 0.027636731 |
| GO:0002376 | immune system process | 1019 | 0.625942135 | 0.590968126 | 0.014635099 |
| GO:0007398 | ectoderm development | 560 | 0.626508768 | 0.56999193 | 0.020515783 |
| GO:0000398 | mRNA splicing, via spliceosome | 344 | 0.626629095 | 0.602907288 | 0.022438555 |
| GO:0005739 | mitochondrion | 64 | 0.627383016 | 0.644679844 | 0.04763292 |
| GO:0001501 | skeletal system development | 72 | 0.628571429 | 0.550598911 | 0.057162095 |
| GO:0048731 | system development | 667 | 0.629643742 | 0.569282105 | 0.016780603 |
| GO:0009987 | cellular process | 3209 | 0.629867207 | 0.553053067 | 0.008205547 |
| GO:0005743 | mitochondrial inner membrane | 58 | 0.630057803 | 0.643071858 | 0.056957086 |
| GO:0006886 | intracellular protein transport | 833 | 0.631972318 | 0.574894493 | 0.01568653 |
| GO:0015031 | protein transport | 833 | 0.631972318 | 0.579204231 | 0.01640927 |
| GO:0006259 | DNA metabolic process | 329 | 0.632155145 | 0.573486694 | 0.025416888 |
| GO:0016301 | kinase activity | 1416 | 0.633410474 | 0.553227775 | 0.012313849 |
| GO:0006139 | nucleobase-containing compound metabolic process | 1835 | 0.634624116 | 0.587929306 | 0.009768593 |
| GO:0006091 | generation of precursor metabolites and energy | 162 | 0.634887006 | 0.684702737 | 0.034744346 |
| GO:0006928 | obsolete movement of cell or subcellular component | 238 | 0.635476464 | 0.570985452 | 0.030540153 |
| GO:0003729 | mRNA binding | 310 | 0.636867089 | 0.598562152 | 0.027403299 |
| GO:0050896 | response to stimulus | 780 | 0.637687897 | 0.586646382 | 0.015916131 |
| GO:0005085 | guanyl-nucleotide exchange factor activity | 59 | 0.637964775 | 0.541510025 | 0.069671912 |
| GO:0030234 | enzyme regulator activity | 739 | 0.637994554 | 0.564430286 | 0.015456359 |
| GO:0006521 | regulation of cellular amino acid metabolic process | 23 | 0.63800905 | 0.586640862 | 0.097560477 |
| GO:0016209 | antioxidant activity | 30 | 0.63800905 | 0.639516916 | 0.080056556 |
| GO:0006468 | protein phosphorylation | 1366 | 0.638586001 | 0.55240614 | 0.013950664 |
| GO:0003723 | RNA binding | 413 | 0.638662546 | 0.607727674 | 0.023955671 |
| GO:0008017 | microtubule binding | 218 | 0.639439907 | 0.548167785 | 0.033573039 |
| GO:0004672 | protein kinase activity | 1097 | 0.641737971 | 0.551450737 | 0.012607221 |
| GO:0006350 | DNA-templated transcription | 750 | 0.642010264 | 0.585216082 | 0.016311325 |
| GO:0006366 | transcription by RNA polymerase II | 748 | 0.642010264 | 0.585455612 | 0.015931733 |
| GO:0009790 | embryo development | 107 | 0.643312102 | 0.620879244 | 0.042445539 |
| GO:0006936 | muscle contraction | 345 | 0.643379663 | 0.539140067 | 0.025508571 |
| GO:0005488 | binding | 2914 | 0.643445487 | 0.586063913 | 0.007077006 |
| GO:0005996 | monosaccharide metabolic process | 189 | 0.643925793 | 0.60367787 | 0.033296478 |
| GO:0003678 | DNA helicase activity | 55 | 0.644 | 0.572272561 | 0.062714033 |
| GO:0007059 | chromosome segregation | 260 | 0.644465291 | 0.568690153 | 0.029326777 |
| GO:0006915 | apoptotic process | 403 | 0.646470956 | 0.60875029 | 0.023826666 |
| GO:0003676 | nucleic acid binding | 1677 | 0.647835769 | 0.60293455 | 0.011058052 |
| GO:0032502 | developmental process | 1106 | 0.648040224 | 0.561716062 | 0.013519741 |
| GO:0008135 | translation factor activity, RNA binding | 132 | 0.648330059 | 0.648874618 | 0.039457361 |
| GO:0016192 | vesicle-mediated transport | 575 | 0.648353828 | 0.569967866 | 0.017890797 |
| GO:0007389 | pattern specification process | 91 | 0.65 | 0.613883425 | 0.053042646 |
| GO:0004857 | enzyme inhibitor activity | 270 | 0.650512582 | 0.588360979 | 0.027872006 |
| GO:0006605 | protein targeting | 80 | 0.651669086 | 0.594734251 | 0.047966544 |
| GO:0006897 | endocytosis | 229 | 0.652881041 | 0.572441049 | 0.025374002 |
| GO:0003677 | DNA binding | 687 | 0.653489235 | 0.588190529 | 0.017724067 |
| GO:0007254 | JNK cascade | 82 | 0.654371585 | 0.597094374 | 0.056518742 |
| GO:0007242 | intracellular signal transduction | 1223 | 0.655252692 | 0.571797339 | 0.013283839 |
| GO:0005634 | nucleus | 21 | 0.655629139 | 0.641556633 | 0.109531348 |
| GO:0016070 | RNA metabolic process | 661 | 0.655755793 | 0.595499431 | 0.018422415 |
| GO:0019722 | calcium-mediated signaling | 458 | 0.656634747 | 0.565104404 | 0.023014894 |
| GO:0006397 | mRNA processing | 414 | 0.658679135 | 0.606653083 | 0.019273936 |
| GO:0006950 | response to stress | 605 | 0.658863545 | 0.582334583 | 0.01849788 |
| GO:0005886 | plasma membrane | 102 | 0.65902965 | 0.484724296 | 0.046112135 |
| GO:0000003 | reproduction | 272 | 0.659881255 | 0.574384954 | 0.029189579 |
| GO:0042116 | macrophage activation | 41 | 0.663120567 | 0.635033595 | 0.078680819 |
| GO:0019208 | phosphatase regulator activity | 44 | 0.663529412 | 0.583530722 | 0.066249604 |
| GO:0007276 | gamete generation | 269 | 0.663632423 | 0.567589609 | 0.024089349 |
| GO:0043066 | negative regulation of apoptotic process | 200 | 0.66383257 | 0.615260605 | 0.030765342 |
| GO:0005102 | signaling receptor binding | 167 | 0.664217487 | 0.625177349 | 0.031282425 |
| GO:0005083 | GTPase regulator activity | 165 | 0.66572836 | 0.550481345 | 0.034076465 |
| GO:0004715 | non-membrane spanning protein tyrosine kinase activity | 56 | 0.666666667 | 0.580129178 | 0.061943153 |
| GO:0005874 | microtubule | 473 | 0.666666667 | 0.552551454 | 0.02115745 |
| GO:0030528 | obsolete transcription regulator activity | 664 | 0.667376161 | 0.569787562 | 0.017974191 |
| GO:0005516 | calmodulin binding | 296 | 0.667892157 | 0.592190768 | 0.026051485 |
| GO:0030879 | mammary gland development | 60 | 0.668449198 | 0.545533465 | 0.060299602 |
| GO:0005509 | calcium ion binding | 368 | 0.670177749 | 0.6006682 | 0.022485066 |
| GO:0003756 | protein disulfide isomerase activity | 28 | 0.671641791 | 0.625221997 | 0.086808597 |
| GO:0005737 | cytoplasm | 108 | 0.671693735 | 0.652497429 | 0.041111563 |
| GO:0003700 | DNA-binding transcription factor activity | 576 | 0.672058498 | 0.580007643 | 0.020033713 |
| GO:0003697 | single-stranded DNA binding | 46 | 0.672605791 | 0.66015194 | 0.053275155 |
| GO:0005622 | intracellular anatomical structure | 836 | 0.672727273 | 0.573408739 | 0.015392888 |
| GO:0009653 | anatomical structure morphogenesis | 575 | 0.672905332 | 0.550898962 | 0.018531763 |
| GO:0032989 | cellular component morphogenesis | 575 | 0.672905332 | 0.549167959 | 0.019252309 |
| GO:0016043 | cellular component organization | 723 | 0.673095945 | 0.561060783 | 0.016167764 |
| GO:0006096 | glycolytic process | 86 | 0.673563218 | 0.615155295 | 0.041819695 |
| GO:0005200 | structural constituent of cytoskeleton | 606 | 0.675084175 | 0.56576527 | 0.017577759 |
| GO:0005856 | cytoskeleton | 606 | 0.675084175 | 0.562421312 | 0.020514749 |
| GO:0006996 | organelle organization | 150 | 0.675675676 | 0.592395598 | 0.032880664 |
| GO:0015629 | actin cytoskeleton | 77 | 0.676748582 | 0.600207559 | 0.053447527 |
| GO:0006325 | chromatin organization | 137 | 0.678414097 | 0.592152793 | 0.037716483 |
| GO:0007498 | mesoderm development | 230 | 0.682310469 | 0.568973715 | 0.032128962 |
| GO:0006119 | oxidative phosphorylation | 33 | 0.682432432 | 0.658503604 | 0.077379535 |
| GO:0006357 | regulation of transcription by RNA polymerase II | 433 | 0.683658171 | 0.593044021 | 0.022965885 |
| GO:0005976 | polysaccharide metabolic process | 121 | 0.684322034 | 0.6028503 | 0.040438251 |
| GO:0006206 | pyrimidine nucleobase metabolic process | 45 | 0.684863524 | 0.6276473 | 0.066472265 |
| GO:0006094 | gluconeogenesis | 63 | 0.687022901 | 0.564642647 | 0.055201761 |
| GO:0016079 | synaptic vesicle exocytosis | 23 | 0.687782805 | 0.599492386 | 0.098472215 |
| GO:0003688 | DNA replication origin binding | 24 | 0.688 | 0.627093588 | 0.081445227 |
| GO:0007049 | cell cycle | 1439 | 0.689782217 | 0.57040146 | 0.010824217 |
| GO:0003682 | chromatin binding | 110 | 0.690402477 | 0.587713001 | 0.045959412 |
| GO:0006367 | transcription initiation from RNA polymerase II promoter | 24 | 0.692307692 | 0.52697699 | 0.112649521 |
| GO:0003777 | microtubule motor activity | 103 | 0.692961165 | 0.560720653 | 0.051186746 |
| GO:0000165 | MAPK cascade | 338 | 0.693843031 | 0.588014774 | 0.029385626 |
| GO:0007126 | meiotic cell cycle | 276 | 0.696105982 | 0.574456573 | 0.027446769 |
| GO:0007283 | spermatogenesis | 179 | 0.697766097 | 0.559780531 | 0.031268274 |
| GO:0005544 | calcium-dependent phospholipid binding | 106 | 0.698765432 | 0.563975915 | 0.045044897 |
| GO:0007379 | segment specification | 43 | 0.704607046 | 0.586684519 | 0.071930346 |
| GO:0007067 | mitotic cell cycle | 587 | 0.70764526 | 0.573984693 | 0.019575286 |
| GO:0006378 | mRNA polyadenylation | 39 | 0.710743802 | 0.640230117 | 0.071484456 |
| GO:0008143 | poly(A) binding | 39 | 0.710743802 | 0.641022723 | 0.0655932 |
| GO:0031124 | mRNA 3'-end processing | 41 | 0.710743802 | 0.63981978 | 0.067402329 |
| GO:0016791 | phosphatase activity | 198 | 0.712999437 | 0.608024257 | 0.035778963 |
| GO:0003774 | cytoskeletal motor activity | 120 | 0.715351812 | 0.548914459 | 0.04606166 |
| GO:0006412 | translation | 382 | 0.715654342 | 0.683314784 | 0.021696982 |
| GO:0005198 | structural molecule activity | 831 | 0.716534141 | 0.60439391 | 0.015125542 |
| GO:0000910 | cytokinesis | 201 | 0.738547486 | 0.554193399 | 0.031553981 |
| GO:0006099 | tricarboxylic acid cycle | 32 | 0.739776952 | 0.744715951 | 0.073005605 |
| GO:0006800 | obsolete oxygen and reactive oxygen species metabolic process | 67 | 0.740740741 | 0.672346057 | 0.067736736 |
| GO:0016072 | rRNA metabolic process | 179 | 0.741491673 | 0.596550886 | 0.035791514 |
| GO:0005977 | glycogen metabolic process | 99 | 0.741697417 | 0.617370022 | 0.046489737 |
| GO:0004601 | peroxidase activity | 28 | 0.748663102 | 0.675695418 | 0.099283285 |
| GO:0019209 | kinase activator activity | 44 | 0.753117207 | 0.561002301 | 0.0643505 |
| GO:0004721 | phosphoprotein phosphatase activity | 171 | 0.753716871 | 0.610050505 | 0.030731241 |
| GO:0008047 | enzyme activator activity | 50 | 0.759036145 | 0.589251494 | 0.066961541 |
| GO:0006461 | protein-containing complex assembly | 58 | 0.762781186 | 0.692280337 | 0.05406727 |
| GO:0030529 | ribonucleoprotein complex | 76 | 0.773349938 | 0.64380531 | 0.045346437 |
| GO:0019201 | nucleoside monophosphate kinase activity | 40 | 0.776978417 | 0.640616763 | 0.082829792 |
| GO:0006144 | purine nucleobase metabolic process | 167 | 0.791492911 | 0.620143659 | 0.036017127 |
| GO:0006369 | termination of RNA polymerase II transcription | 112 | 0.798029557 | 0.570815493 | 0.046496402 |
| GO:0019212 | phosphatase inhibitor activity | 21 | 0.81 | 0.590968236 | 0.100055853 |
| GO:0030120 | vesicle coat | 33 | 0.811965812 | 0.643773029 | 0.082904072 |
| GO:0006109 | regulation of carbohydrate metabolic process | 34 | 0.813688213 | 0.662244692 | 0.075235856 |
| GO:0007517 | muscle organ development | 26 | 0.837662338 | 0.556565657 | 0.099596622 |
| GO:0003735 | structural constituent of ribosome | 213 | 0.853010164 | 0.778815619 | 0.026152334 |
| GO:0015078 | proton transmembrane transporter activity | 31 | 0.855072464 | 0.742446615 | 0.063812089 |
| GO:0019219 | regulation of nucleobase-containing compound metabolic process | 31 | 0.878661088 | 0.657460317 | 0.084951587 |

## **Table S3**. Probability of post-WGD gene loss in a given *Paramecium* as a function of retention and expression level conservation of the orthologous genes in the other *Paramecium* species.

| Species #1 | Species #2 | Probability of post-WGD loss in species #2 conditional on retention in species #1 | Probability of post-WGD loss in species #2 conditional on retention but divergent expression level in species #1 | p-value^*^ | Fraction of lost genes in species #2 that were orthologous to the copy with the lowest expression in species #1 | p-value^*^ |
| --- | --- | --- | --- | --- | --- | --- |
| *P. biaurelia* | *P. decaurelia* | 0.111 | 0.384 | 7.3E-51 | 0.717 | 6.7E-33 |
| *P. biaurelia* | *P. dodecaurelia* | 0.103 | 0.347 | 4.9E-44 | 0.705 | 1.1E-27 |
| *P. biaurelia* | *P. jennignsi* | 0.176 | 0.308 | 9.4E-8 | 0.523 | 1.6E-1 |
| *P. biaurelia* | *P. novaurelia* | 0.131 | 0.372 | 1.2E-32 | 0.707 | 2.5E-32 |
| *P. biaurelia* | *P. octaurelia* | 0.128 | 0.412 | 8.7E-51 | 0.684 | 9.4E-29 |
| *P. biaurelia* | *P. pentaurelia* | 0.098 | 0.313 | 4.7E-37 | 0.710 | 3.7E-28 |
| *P. biaurelia* | *P. primaurelia* | 0.157 | 0.461 | 9.7E-47 | 0.675 | 1.8E-29 |
| *P. biaurelia* | *P. quadecaurelia* | 0.287 | 0.601 | 8.1E-31 | 0.671 | 2.4E-45 |
| *P. biaurelia* | *P. sexaurelia* | 0.348 | 0.629 | 5.1E-26 | 0.520 | 5.1E-2 |
| *P. biaurelia* | *P. sonneborni* | 0.143 | 0.308 | 4.5E-17 | 0.495 | 8.0E-1 |
| *P. biaurelia* | *P. tetraurelia* | 0.123 | 0.413 | 3.2E-55 | 0.710 | 8.6E-36 |
| *P. biaurelia* | *P. tredecaurelia* | 0.310 | 0.620 | 4.5E-33 | 0.659 | 9.2E-49 |
| *P. decaurelia* | *P. biaurelia* | 0.098 | 0.270 | 9.3E-24 | 0.709 | 2.7E-27 |
| *P. decaurelia* | *P. dodecaurelia* | 0.031 | 0.225 | 9.6E-72 | 0.817 | 4.5E-21 |
| *P. decaurelia* | *P. jennignsi* | 0.168 | 0.274 | 1.4E-5 | 0.494 | 7.3E-1 |
| *P. decaurelia* | *P. novaurelia* | 0.123 | 0.291 | 2.5E-17 | 0.698 | 5.8E-28 |
| *P. decaurelia* | *P. octaurelia* | 0.056 | 0.285 | 3.0E-64 | 0.772 | 1.2E-27 |
| *P. decaurelia* | *P. pentaurelia* | 0.092 | 0.242 | 6.4E-20 | 0.697 | 2.8E-23 |
| *P. decaurelia* | *P. primaurelia* | 0.151 | 0.347 | 1.1E-20 | 0.667 | 7.4E-26 |
| *P. decaurelia* | *P. quadecaurelia* | 0.274 | 0.443 | 3.3E-10 | 0.671 | 8.7E-44 |
| *P. decaurelia* | *P. sexaurelia* | 0.348 | 0.507 | 3.5E-9 | 0.520 | 5.8E-2 |
| *P. decaurelia* | *P. sonneborni* | 0.139 | 0.267 | 6.7E-11 | 0.516 | 3.4E-1 |
| *P. decaurelia* | *P. tetraurelia* | 0.054 | 0.321 | 3.5E-88 | 0.847 | 1.9E-42 |
| *P. decaurelia* | *P. tredecaurelia* | 0.302 | 0.516 | 1.2E-16 | 0.677 | 2.1E-58 |
| *P. dodecaurelia* | *P. biaurelia* | 0.100 | 0.271 | 3.1E-23 | 0.684 | 2.6E-22 |
| *P. dodecaurelia* | *P. decaurelia* | 0.049 | 0.224 | 4.5E-44 | 0.764 | 6.2E-23 |
| *P. dodecaurelia* | *P. jennignsi* | 0.175 | 0.328 | 5.2E-10 | 0.497 | 8.7E-1 |
| *P. dodecaurelia* | *P. novaurelia* | 0.125 | 0.314 | 7.7E-22 | 0.681 | 3.5E-24 |
| *P. dodecaurelia* | *P. octaurelia* | 0.065 | 0.293 | 9.2E-58 | 0.767 | 2.6E-31 |
| *P. dodecaurelia* | *P. pentaurelia* | 0.097 | 0.263 | 3.2E-23 | 0.677 | 1.9E-20 |
| *P. dodecaurelia* | *P. primaurelia* | 0.157 | 0.370 | 3.2E-24 | 0.648 | 1.2E-21 |
| *P. dodecaurelia* | *P. quadecaurelia* | 0.276 | 0.464 | 1.6E-12 | 0.656 | 1.1E-37 |
| *P. dodecaurelia* | *P. sexaurelia* | 0.354 | 0.525 | 1.8E-10 | 0.522 | 3.2E-2 |
| *P. dodecaurelia* | *P. sonneborni* | 0.143 | 0.276 | 9.4E-12 | 0.496 | 8.0E-1 |
| *P. dodecaurelia* | *P. tetraurelia* | 0.062 | 0.339 | 1.9E-86 | 0.811 | 2.7E-40 |
| *P. dodecaurelia* | *P. tredecaurelia* | 0.308 | 0.504 | 2.6E-14 | 0.647 | 3.3E-42 |
| *P. jennignsi* | *P. biaurelia* | 0.294 | 0.537 | 3.1E-19 | 0.498 | 8.5E-1 |
| *P. jennignsi* | *P. decaurelia* | 0.310 | 0.597 | 5.7E-26 | 0.512 | 2.9E-1 |
| *P. jennignsi* | *P. dodecaurelia* | 0.306 | 0.578 | 9.4E-24 | 0.513 | 2.6E-1 |
| *P. jennignsi* | *P. novaurelia* | 0.305 | 0.603 | 2.0E-25 | 0.521 | 8.3E-2 |
| *P. jennignsi* | *P. octaurelia* | 0.336 | 0.629 | 4.7E-27 | 0.497 | 8.3E-1 |
| *P. jennignsi* | *P. pentaurelia* | 0.302 | 0.566 | 7.2E-23 | 0.502 | 8.6E-1 |
| *P. jennignsi* | *P. primaurelia* | 0.332 | 0.628 | 2.8E-25 | 0.500 | 1.0E+0 |
| *P. jennignsi* | *P. quadecaurelia* | 0.427 | 0.715 | 1.0E-20 | 0.501 | 9.3E-1 |
| *P. jennignsi* | *P. sexaurelia* | 0.420 | 0.730 | 1.5E-27 | 0.512 | 2.3E-1 |
| *P. jennignsi* | *P. sonneborni* | 0.102 | 0.406 | 3.8E-64 | 0.778 | 2.1E-48 |
| *P. jennignsi* | *P. tetraurelia* | 0.333 | 0.620 | 2.2E-26 | 0.507 | 5.1E-1 |
| *P. jennignsi* | *P. tredecaurelia* | 0.464 | 0.741 | 3.1E-21 | 0.504 | 6.7E-1 |
| *P. novaurelia* | *P. biaurelia* | 0.102 | 0.332 | 7.8E-35 | 0.709 | 1.5E-25 |
| *P. novaurelia* | *P. decaurelia* | 0.106 | 0.339 | 1.0E-34 | 0.711 | 6.7E-27 |
| *P. novaurelia* | *P. dodecaurelia* | 0.101 | 0.333 | 5.2E-36 | 0.717 | 2.4E-27 |
| *P. novaurelia* | *P. jennignsi* | 0.165 | 0.311 | 1.2E-8 | 0.519 | 3.1E-1 |
| *P. novaurelia* | *P. octaurelia* | 0.123 | 0.397 | 4.6E-43 | 0.714 | 2.1E-32 |
| *P. novaurelia* | *P. pentaurelia* | 0.071 | 0.244 | 6.6E-28 | 0.707 | 4.4E-18 |
| *P. novaurelia* | *P. primaurelia* | 0.136 | 0.390 | 1.7E-32 | 0.663 | 2.6E-20 |
| *P. novaurelia* | *P. quadecaurelia* | 0.245 | 0.572 | 1.4E-32 | 0.730 | 2.6E-63 |
| *P. novaurelia* | *P. sexaurelia* | 0.341 | 0.538 | 3.9E-12 | 0.512 | 3.1E-1 |
| *P. novaurelia* | *P. sonneborni* | 0.123 | 0.246 | 5.6E-10 | 0.516 | 3.8E-1 |
| *P. novaurelia* | *P. tetraurelia* | 0.121 | 0.376 | 1.3E-38 | 0.718 | 1.8E-33 |
| *P. novaurelia* | *P. tredecaurelia* | 0.271 | 0.623 | 1.0E-39 | 0.738 | 2.5E-83 |
| *P. octaurelia* | *P. biaurelia* | 0.090 | 0.251 | 2.3E-22 | 0.688 | 1.5E-20 |
| *P. octaurelia* | *P. decaurelia* | 0.034 | 0.178 | 7.7E-40 | 0.768 | 2.7E-16 |
| *P. octaurelia* | *P. dodecaurelia* | 0.028 | 0.186 | 2.2E-55 | 0.734 | 2.2E-10 |
| *P. octaurelia* | *P. jennignsi* | 0.165 | 0.259 | 1.3E-4 | 0.509 | 6.3E-1 |
| *P. octaurelia* | *P. novaurelia* | 0.114 | 0.250 | 1.1E-12 | 0.656 | 1.8E-16 |
| *P. octaurelia* | *P. pentaurelia* | 0.082 | 0.231 | 1.2E-21 | 0.659 | 3.2E-14 |
| *P. octaurelia* | *P. primaurelia* | 0.143 | 0.373 | 2.1E-29 | 0.640 | 1.1E-17 |
| *P. octaurelia* | *P. quadecaurelia* | 0.263 | 0.440 | 2.3E-11 | 0.637 | 1.2E-27 |
| *P. octaurelia* | *P. sexaurelia* | 0.338 | 0.528 | 1.1E-12 | 0.518 | 8.3E-2 |
| *P. octaurelia* | *P. sonneborni* | 0.136 | 0.300 | 2.7E-17 | 0.519 | 2.5E-1 |
| *P. octaurelia* | *P. tetraurelia* | 0.033 | 0.270 | 6.8E-102 | 0.852 | 4.1E-27 |
| *P. octaurelia* | *P. tredecaurelia* | 0.288 | 0.529 | 2.0E-21 | 0.640 | 2.0E-35 |
| *P. pentaurelia* | *P. biaurelia* | 0.104 | 0.299 | 1.5E-29 | 0.758 | 3.2E-45 |
| *P. pentaurelia* | *P. decaurelia* | 0.112 | 0.351 | 2.0E-40 | 0.733 | 1.1E-39 |
| *P. pentaurelia* | *P. dodecaurelia* | 0.106 | 0.351 | 6.0E-45 | 0.739 | 1.2E-39 |
| *P. pentaurelia* | *P. jennignsi* | 0.172 | 0.323 | 4.1E-10 | 0.530 | 7.5E-2 |
| *P. pentaurelia* | *P. novaurelia* | 0.109 | 0.374 | 6.3E-46 | 0.757 | 2.2E-42 |
| *P. pentaurelia* | *P. octaurelia* | 0.128 | 0.398 | 4.0E-48 | 0.725 | 5.5E-44 |
| *P. pentaurelia* | *P. primaurelia* | 0.110 | 0.615 | 7.4E-161 | 0.768 | 2.1E-50 |
| *P. pentaurelia* | *P. quadecaurelia* | 0.275 | 0.608 | 3.7E-36 | 0.736 | 2.4E-84 |
| *P. pentaurelia* | *P. sexaurelia* | 0.350 | 0.631 | 1.0E-26 | 0.511 | 2.6E-1 |
| *P. pentaurelia* | *P. sonneborni* | 0.136 | 0.310 | 6.1E-20 | 0.531 | 5.6E-2 |
| *P. pentaurelia* | *P. tetraurelia* | 0.129 | 0.410 | 5.5E-52 | 0.751 | 3.2E-55 |
| *P. pentaurelia* | *P. tredecaurelia* | 0.300 | 0.646 | 1.0E-42 | 0.737 | 8.7E-107 |
| *P. primaurelia* | *P. biaurelia* | 0.090 | 0.141 | 2.8E-3 | 0.610 | 1.9E-6 |
| *P. primaurelia* | *P. decaurelia* | 0.092 | 0.182 | 2.6E-7 | 0.702 | 4.6E-18 |
| *P. primaurelia* | *P. dodecaurelia* | 0.087 | 0.194 | 4.2E-10 | 0.681 | 1.7E-14 |
| *P. primaurelia* | *P. jennignsi* | 0.155 | 0.201 | 7.1E-2 | 0.512 | 5.7E-1 |
| *P. primaurelia* | *P. novaurelia* | 0.091 | 0.196 | 1.1E-8 | 0.709 | 8.4E-18 |
| *P. primaurelia* | *P. octaurelia* | 0.111 | 0.213 | 4.7E-8 | 0.660 | 4.2E-15 |
| *P. primaurelia* | *P. pentaurelia* | 0.023 | 0.047 | 1.0E-2 | 0.647 | 1.8E-3 |
| *P. primaurelia* | *P. quadecaurelia* | 0.243 | 0.357 | 3.1E-5 | 0.631 | 1.0E-18 |
| *P. primaurelia* | *P. sexaurelia* | 0.316 | 0.384 | 1.7E-2 | 0.502 | 8.8E-1 |
| *P. primaurelia* | *P. sonneborni* | 0.114 | 0.162 | 1.3E-2 | 0.502 | 9.7E-1 |
| *P. primaurelia* | *P. tetraurelia* | 0.106 | 0.207 | 2.5E-8 | 0.692 | 3.8E-20 |
| *P. primaurelia* | *P. tredecaurelia* | 0.271 | 0.369 | 2.2E-4 | 0.633 | 7.9E-24 |
| *P. quadecaurelia* | *P. biaurelia* | 0.077 | 0.154 | 4.1E-5 | 0.636 | 3.2E-7 |
| *P. quadecaurelia* | *P. decaurelia* | 0.067 | 0.137 | 8.5E-5 | 0.616 | 5.0E-5 |
| *P. quadecaurelia* | *P. dodecaurelia* | 0.063 | 0.105 | 1.5E-2 | 0.627 | 1.5E-5 |
| *P. quadecaurelia* | *P. jennignsi* | 0.134 | 0.232 | 3.3E-4 | 0.537 | 1.1E-1 |
| *P. quadecaurelia* | *P. novaurelia* | 0.046 | 0.098 | 9.7E-4 | 0.614 | 1.7E-3 |
| *P. quadecaurelia* | *P. octaurelia* | 0.078 | 0.166 | 2.4E-6 | 0.608 | 3.1E-5 |
| *P. quadecaurelia* | *P. pentaurelia* | 0.048 | 0.080 | 4.1E-2 | 0.583 | 1.5E-2 |
| *P. quadecaurelia* | *P. primaurelia* | 0.096 | 0.168 | 6.5E-4 | 0.618 | 1.0E-6 |
| *P. quadecaurelia* | *P. sexaurelia* | 0.285 | 0.416 | 3.0E-5 | 0.517 | 2.4E-1 |
| *P. quadecaurelia* | *P. sonneborni* | 0.095 | 0.152 | 5.2E-3 | 0.499 | 1.0E+0 |
| *P. quadecaurelia* | *P. tetraurelia* | 0.073 | 0.152 | 1.0E-5 | 0.625 | 3.0E-6 |
| *P. quadecaurelia* | *P. tredecaurelia* | 0.120 | 0.297 | 2.6E-15 | 0.735 | 3.0E-29 |
| *P. sexaurelia* | *P. biaurelia* | 0.203 | 0.355 | 1.9E-9 | 0.501 | 9.8E-1 |
| *P. sexaurelia* | *P. decaurelia* | 0.214 | 0.325 | 1.6E-5 | 0.489 | 4.5E-1 |
| *P. sexaurelia* | *P. dodecaurelia* | 0.213 | 0.357 | 1.7E-8 | 0.493 | 6.4E-1 |
| *P. sexaurelia* | *P. jennignsi* | 0.154 | 0.205 | 5.4E-2 | 0.504 | 8.8E-1 |
| *P. sexaurelia* | *P. novaurelia* | 0.208 | 0.341 | 7.9E-7 | 0.495 | 7.8E-1 |
| *P. sexaurelia* | *P. octaurelia* | 0.233 | 0.374 | 5.2E-8 | 0.485 | 2.7E-1 |
| *P. sexaurelia* | *P. pentaurelia* | 0.207 | 0.371 | 4.0E-11 | 0.500 | 1.0E+0 |
| *P. sexaurelia* | *P. primaurelia* | 0.230 | 0.422 | 7.1E-13 | 0.494 | 6.7E-1 |
| *P. sexaurelia* | *P. quadecaurelia* | 0.330 | 0.514 | 3.3E-9 | 0.485 | 2.2E-1 |
| *P. sexaurelia* | *P. sonneborni* | 0.123 | 0.211 | 1.1E-5 | 0.514 | 4.8E-1 |
| *P. sexaurelia* | *P. tetraurelia* | 0.230 | 0.384 | 1.7E-9 | 0.502 | 8.9E-1 |
| *P. sexaurelia* | *P. tredecaurelia* | 0.356 | 0.541 | 4.6E-10 | 0.493 | 5.3E-1 |
| *P. sonneborni* | *P. biaurelia* | 0.301 | 0.524 | 2.3E-22 | 0.513 | 1.8E-1 |
| *P. sonneborni* | *P. decaurelia* | 0.316 | 0.584 | 2.8E-31 | 0.504 | 7.2E-1 |
| *P. sonneborni* | *P. dodecaurelia* | 0.311 | 0.563 | 2.6E-28 | 0.505 | 6.5E-1 |
| *P. sonneborni* | *P. jennignsi* | 0.131 | 0.311 | 4.5E-21 | 0.711 | 2.7E-37 |
| *P. sonneborni* | *P. novaurelia* | 0.306 | 0.565 | 1.2E-26 | 0.523 | 2.3E-2 |
| *P. sonneborni* | *P. octaurelia* | 0.341 | 0.623 | 8.6E-35 | 0.503 | 7.6E-1 |
| *P. sonneborni* | *P. pentaurelia* | 0.306 | 0.556 | 1.2E-28 | 0.509 | 3.6E-1 |
| *P. sonneborni* | *P. primaurelia* | 0.330 | 0.614 | 2.0E-32 | 0.500 | 9.8E-1 |
| *P. sonneborni* | *P. quadecaurelia* | 0.436 | 0.714 | 3.7E-26 | 0.502 | 7.9E-1 |
| *P. sonneborni* | *P. sexaurelia* | 0.424 | 0.731 | 9.6E-38 | 0.508 | 3.1E-1 |
| *P. sonneborni* | *P. tetraurelia* | 0.337 | 0.622 | 1.0E-35 | 0.505 | 6.0E-1 |
| *P. sonneborni* | *P. tredecaurelia* | 0.460 | 0.751 | 3.7E-32 | 0.511 | 1.9E-1 |
| *P. tetraurelia* | *P. biaurelia* | 0.092 | 0.232 | 1.4E-17 | 0.699 | 8.0E-24 |
| *P. tetraurelia* | *P. decaurelia* | 0.041 | 0.236 | 2.1E-60 | 0.770 | 1.6E-19 |
| *P. tetraurelia* | *P. dodecaurelia* | 0.032 | 0.199 | 1.1E-55 | 0.775 | 4.6E-16 |
| *P. tetraurelia* | *P. jennignsi* | 0.166 | 0.284 | 1.1E-6 | 0.512 | 5.0E-1 |
| *P. tetraurelia* | *P. novaurelia* | 0.117 | 0.287 | 8.8E-19 | 0.672 | 2.0E-20 |
| *P. tetraurelia* | *P. octaurelia* | 0.043 | 0.242 | 9.7E-62 | 0.739 | 4.2E-17 |
| *P. tetraurelia* | *P. pentaurelia* | 0.088 | 0.241 | 1.4E-21 | 0.665 | 2.1E-16 |
| *P. tetraurelia* | *P. primaurelia* | 0.147 | 0.381 | 3.3E-30 | 0.638 | 7.9E-18 |
| *P. tetraurelia* | *P. quadecaurelia* | 0.264 | 0.497 | 9.7E-19 | 0.669 | 5.7E-42 |
| *P. tetraurelia* | *P. sexaurelia* | 0.340 | 0.524 | 2.9E-12 | 0.515 | 1.4E-1 |
| *P. tetraurelia* | *P. sonneborni* | 0.137 | 0.269 | 5.5E-12 | 0.515 | 3.7E-1 |
| *P. tetraurelia* | *P. tredecaurelia* | 0.291 | 0.564 | 3.0E-27 | 0.663 | 1.2E-48 |
| *P. tredecaurelia* | *P. biaurelia* | 0.069 | 0.132 | 2.0E-4 | 0.643 | 1.4E-7 |
| *P. tredecaurelia* | *P. decaurelia* | 0.063 | 0.129 | 4.6E-5 | 0.661 | 1.6E-8 |
| *P. tredecaurelia* | *P. dodecaurelia* | 0.059 | 0.117 | 2.7E-4 | 0.664 | 2.3E-8 |
| *P. tredecaurelia* | *P. jennignsi* | 0.142 | 0.197 | 4.5E-2 | 0.528 | 2.1E-1 |
| *P. tredecaurelia* | *P. novaurelia* | 0.044 | 0.094 | 5.4E-4 | 0.655 | 1.9E-5 |
| *P. tredecaurelia* | *P. octaurelia* | 0.070 | 0.170 | 3.6E-9 | 0.614 | 1.7E-5 |
| *P. tredecaurelia* | *P. pentaurelia* | 0.039 | 0.103 | 1.2E-6 | 0.571 | 5.5E-2 |
| *P. tredecaurelia* | *P. primaurelia* | 0.092 | 0.218 | 1.7E-10 | 0.583 | 5.3E-4 |
| *P. tredecaurelia* | *P. quadecaurelia* | 0.094 | 0.233 | 1.2E-11 | 0.704 | 7.2E-17 |
| *P. tredecaurelia* | *P. sexaurelia* | 0.285 | 0.391 | 3.6E-4 | 0.514 | 3.0E-1 |
| *P. tredecaurelia* | *P. sonneborni* | 0.093 | 0.138 | 2.1E-2 | 0.513 | 6.1E-1 |
| *P. tredecaurelia* | *P. tetraurelia* | 0.067 | 0.154 | 1.7E-7 | 0.662 | 2.4E-9 |

* P-values were generated by proportions tests with Yates continuity correction in R.

## **Table S4**. Segmental duplications in Paramecium species.

| Species | # Segmental duplications | Fraction of retained ohnologs involved in a segmental duplication | Fraction of single ohnologs involved in a segmental duplication | P value^*^ |
| --- | --- | --- | --- | --- |
| *P. biaurelia* | 34 | 0.00074 | 0.0017 | 0.016946 |
| *P. decaurelia* | 26 | 0.00035 | 0.0017 | 0.0000845 |
| *P. dodecaurelia* | 31 | 0.00051 | 0.0017 | 0.000984 |
| *P. jenningsi* | 20 | 0.00052 | 0.0012 | 0.100879 |
| *P. novaurelia* | 18 | 0.0004 | 0.0012 | 0.027285 |
| *P. octaurelia* | 21 | 0.00042 | 0.00097 | 0.086093 |
| *P. pentaurelia* | 278 | 0.0065 | 0.011 | 0.000013 |
| *P. primaurelia* | 35 | 0.00082 | 0.0019 | 0.016052 |
| *P. quadecaurelia* | 21 | 0.00045 | 0.0013 | 0.025925 |
| *P. sexaurelia* | 25 | 0.00045 | 0.0012 | 0.023424 |
| *P. tetraurelia* | 52 | 0.0011 | 0.002 | 0.054674 |
| *P. tredecaurelia* | 54 | 0.0012 | 0.0024 | 0.015686 |

* P-values were generated by proportions tests with Yates continuity correction in R.

## **Table S5.** Number of paralogons with K_s_ change points in aurelia species

| Species | # Paralogons with K_s_ cpt(s) | # Paralogons in total | % |
| --- | --- | --- | --- |
| *P. sonneborni* | 9 | 104 | 8.65 |
| *P. sexaurelia* | 3 | 106 | 2.83 |
| *P. jenningsi* | 1 | 228 | 0.44 |
| *P. biaurelia* | 3 | 400 | 0.75 |
| *P. decaurelia* | 1 | 316 | 0.32 |
| *P. dodecaurelia* | 3 | 315 | 0.95 |
| *P. novaurelia* | 3 | 504 | 0.60 |
| *P. octaurelia* | 5 | 120 | 4.17 |
| *P. pentaurelia* | 4 | 106 | 3.77 |
| *P. primaurelia* | 7 | 124 | 5.65 |
| *P. quadecaurelia* | 5 | 247 | 2.02 |
| *P. tetraurelia* | 6 | 131 | 4.58 |
| *P. tredecaurelia* | 7 | 114 | 6.14 |


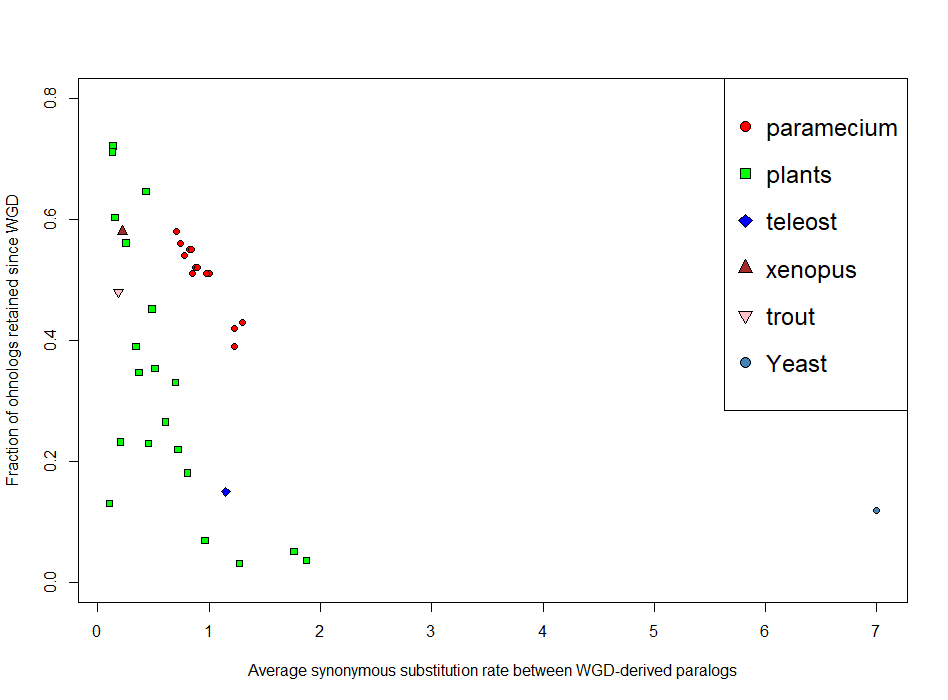


## **Figure S1**. Post-WGD gene retention as a function of sequence divergence (the number of nonsynonymous substitutions per non-synonymous site, *K_s_*) between the remaining pairs of WGD-derived paralogs in *P. aurelia* and other eukaryotes having experienced ancestral WGDs, including yeast (Casola et al. 2012), plants (Ren et al. 2018), teleost (Jaillon et al. 2004), salmonid (Berthelot et al. 2014) and *Xenopus* (Session et al. 2016). See also Figure 2b.


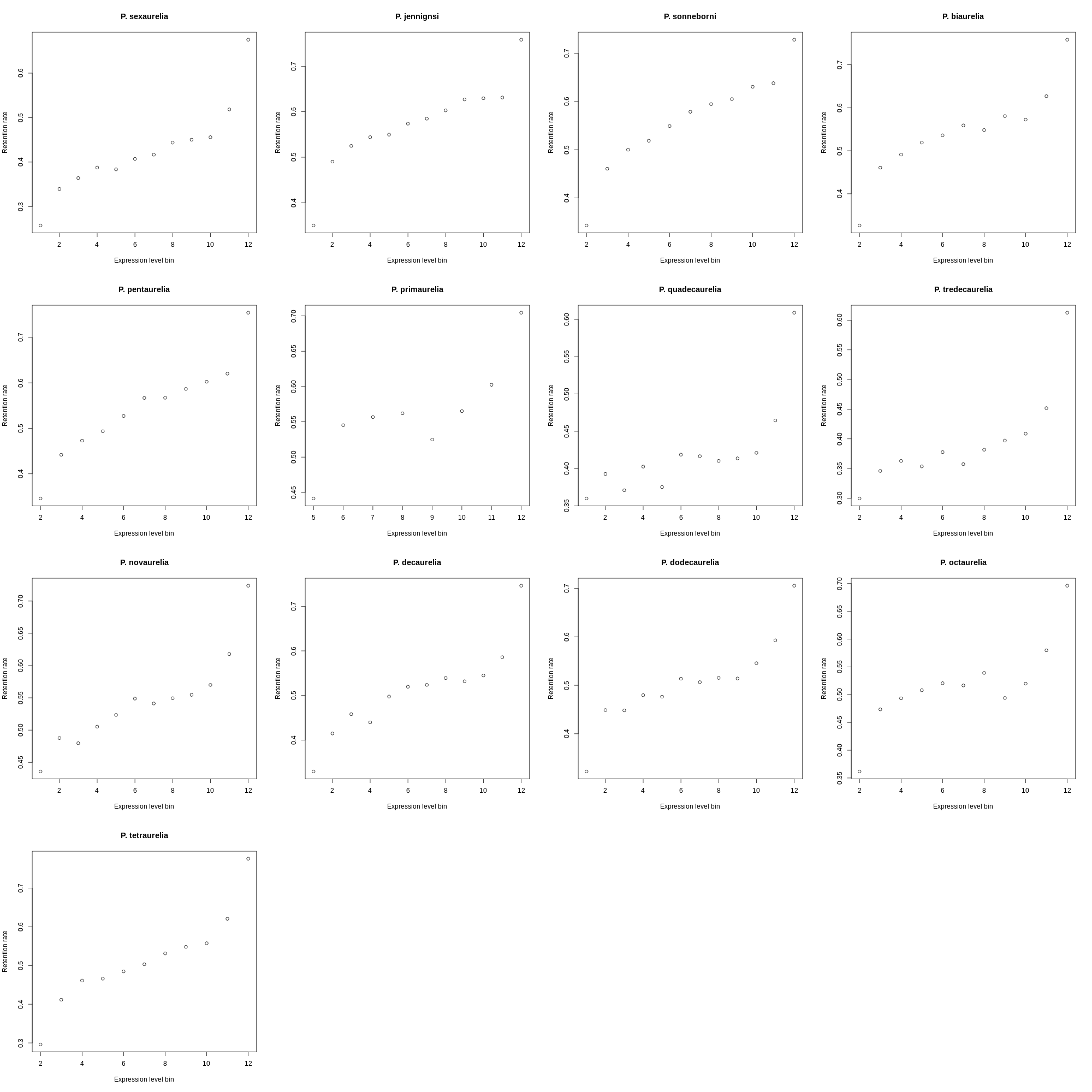


## **Figure S2**. Post-WGD retention rate for genes grouped in bins of expression level (12 bins of equal size) in each *P. aurelia* species.

c)

b)

a)


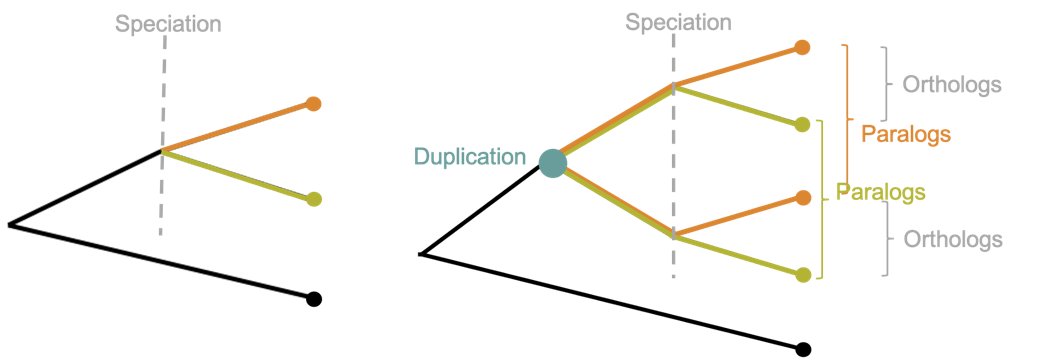

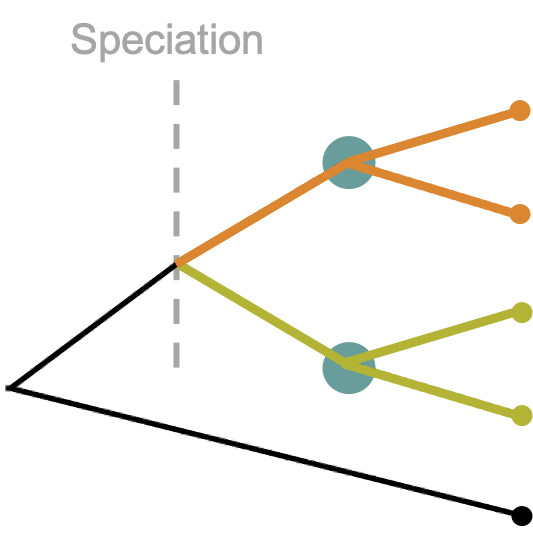


## **Figure S3**. Schematic depiction of the two major subclades in *Paramecium* aurelia, shown as the orange and the green branches. a) The species tree of the two *P. aurelia* subclades and the outgroup. b) One scenario of a gene tree after the whole-genome duplication. c) An alternative hypothesis where two independent WGD events occurred, one in each subclade. The hypothesis was arisen from the observations that in some gene trees, duplicates from the same subclade tend to cluster together.


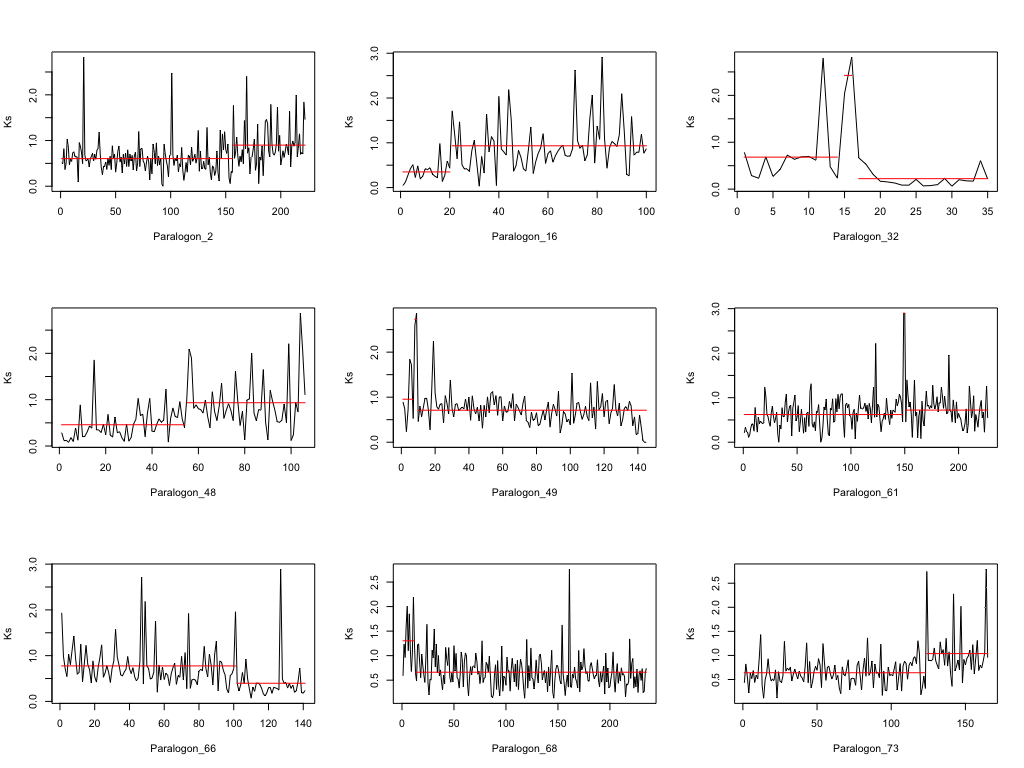


## **Figure S4.** Nine *Paramecium sonneborni* paralogons with change points detected, out of the 104 total paralogons. On the x axis is the gene order along each paralogon. The y axis represents the *Ks* between paralogous gene pairs. Changes in the horizontal red lines indicate significant shifts in *Ks* mean.


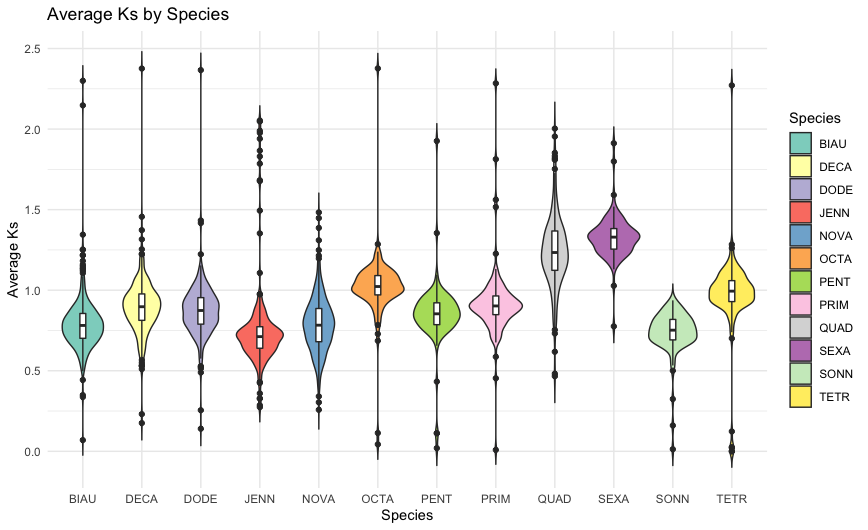


## **Figure S5.** Average synonymous substitution rate (*K_s_*) between paralogous gene pairs for each paralogon created by the most recent WGD in 12 *Paramecium* species.
